# Consensus, cooperative learning, and flocking for multiagent predator avoidance

**DOI:** 10.1177/1729881420960342

**Published:** 2020-09-24

**Authors:** Zachary Young, Hung Manh La

**Affiliations:** Department of Computer Science and Engineering, Advanced Robotics and Automation (ARA) Laboratory, University of Nevada, Reno, NV, USA

**Keywords:** Distributed algorithms for multirobot coordination, mobile robots and multirobot systems, multiagent robot teams, mobile sensor networks, swarm robotics, multiagent learning, function approximation, consensus, flocking control

## Abstract

Multiagent coordination is highly desirable with many uses in a variety of tasks. In nature, the phenomenon of coordinated flocking is highly common with applications related to defending or escaping from predators. In this article, a hybrid multiagent system that integrates consensus, cooperative learning, and flocking control to determine the direction of attacking predators and learns to flock away from them in a coordinated manner is proposed. This system is entirely distributed requiring only communication between neighboring agents. The fusion of consensus and collaborative reinforcement learning allows agents to cooperatively learn in a variety of multiagent coordination tasks, but this article focuses on flocking away from attacking predators. The results of the flocking show that the agents are able to effectively flock to a target without collision with each other or obstacles. Multiple reinforcement learning methods are evaluated for the task with cooperative learning utilizing function approximation for state-space reduction performing the best. The results of the proposed consensus algorithm show that it provides quick and accurate transmission of information between agents in the flock. Simulations are conducted to show and validate the proposed hybrid system in both one and two predator environments, resulting in an efficient cooperative learning behavior. In the future, the system of using consensus to determine the state and reinforcement learning to learn the states can be applied to additional multiagent tasks.

## Introduction

### Motivation

Multiagent cooperative learning has been continuing to be a large research interest in the field of robotics with a wide range of applications.^1–3^ Tracking wildfires using multiple agents communicating together to better handle the fire, so it does not destroy as much, is one such possibility.^4,5^ Another one is that using multiple agents to better map the structure of a pipeline that if it structurally fails could cause large damage and loss of money.^6^ An additional possibility is mapping and exploring unknown environments.^7^ This article aims to solve the task of multiagent predator avoidance^8,9^ with an intelligent hybrid system.

Most current multiagent research incorporates some form of consensus,^10^ movement control,^11^ or reinforcement learning,^12^ but a combination of all three to achieve an efficient cooperative learning behavior is largely unexplored. Some uses of consensus are to determine the location of obstacles^13^ or to make measurements in a scalar field.^14–16^ Movement control of multiagent systems can come in the form of cooperatively doing path planning as in the literature.^17–19^ Alternatively, there are means of control through flocking in varying formations^20–22^ to achieve a variety of tasks.^23,24^ Reinforcement learning has been implemented cooperatively in a variety of ways for multiagent environments, such as a GridWorld^25,26^ and box pushing.^27^ Although all of these use for consensus, movement control, and reinforcement learning that are good in their own right, this article aims to make a more intelligent hybrid system.

In nature, flocking has long been observed in many environments^28–30^ with one possible goal being to defend from predators as can be seen with schools of fish. Even simulated environments^31,32^ that reward individuals for their own survival result in flocking like formations of the agents. It is thus clear that flocking for survival has clear benefits in both natural and simulated environments. The goal of this article is then to create a hybrid system that combines consensus, flocking, and multiagent reinforcement learning into one intelligent system that can sense and learn to escape from attacking predators.

### Literature review

#### Flocking background.

Methods of ensuring agents flocking have been proposed and studied in the literature.^33–37^ Inspired by the natural world of birds and fish flocking together,^38^ flocking algorithms have been formed. The algorithms allow agents to flock in different patterns in a distributed manner that requires on communication between direct neighbors rather than the entire flock. The main rules that define flocking are that flockmates maintain a distance from each other without getting too close or far from each other and match the velocity of the other flockmates. Reinforcement learning approaches have also been applied to flocking in which agents individually or cooperatively learn to flock without the use of specific algorithms.^39,40^ There are also cases in which reinforcement learning is implemented to teach agents to go to specific targets, but in cases where learning fails, a flocking algorithm to avoid obstacles is used.^41^ Flocking by itself is not particularly useful if the agents flock directly to a predator so this work aims to combine learning with flocking to effectively escape from predators. Other flocking implementations such as that proposed in the literature^42^ allow agents to flock together with minimal information transfer, but the formation is not ideal for quickly avoiding a predator. Additional flocking features can be added, such as handling faulty robots,^43^ time delay for information transfer between robots,^44^ or change of formation.^45^ For this article, these are not the focus but could potentially be added in the future.

#### Reinforcement learning background.

Cooperation is an important part of a flock learning to do a task together. In the literature,^46^ agents are not necessarily flocking together, but through cooperation, they are completing their task effectively. Cooperation is already a large part of flocking algorithms so that they can be done in a distributed manner. Cooperation must also be used to effectively learn in a distributed manner. Using simple Q-learning^47^ does not achieve the necessary amounts of cooperation required, so a more cooperative approach^9,48^ is required. Unfortunately, the number of agents increases the state space that grows and requires longer amounts of training to effectively learn to flock together to the same destination. For this, function approximation techniques^49,50^ are useful. However, in current research, function approximation in combination with cooperative learning is largely unexplored.^51,52^

#### Consensus background.

Partial observability of the state space is another issue for purposes of escaping attacking predators. For a school of fish, not all fish will be able to see an attacking predator yet they manage to utilize flocking to maximize their safety anyway. For our agents, this is the same case in that only agents on the outside of a flock will be able to see an oncoming predator. A method of communicating to the other agents that a predator is approaching is thus necessary. This can be seen as a sort of event-triggered consensus such as that proposed in the literature.^53^ Algorithms have been proposed to allow multiagent systems to come to a consensus on measurements from multiple agents that do not necessarily agree with each other.^54,55^ Many uses of consensus, however, are for estimating some kinds of measurements.^56,57^ This work intends to use consensus in combination with reinforcement learning to make a more intelligent system. Some works, such as by Zhang et al.^51^ and Xu et al.,^52^ implement a hybrid system of consensus and reinforcement learning, but they use consensus to determine the global reward of the system. This work uses consensus to determine the state relying on local rewards instead.

### Contributions

**Figure 1.**
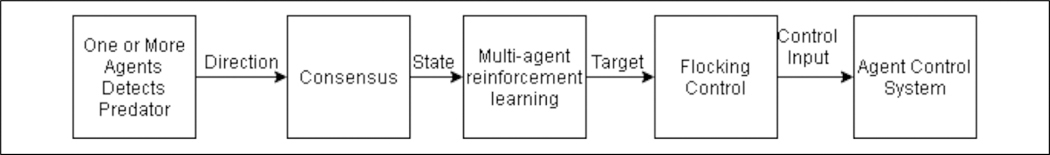
Block diagram of the hybrid system.

In this article, we combine the benefits of consensus, flocking, and reinforcement learning to create a hybrid system, as shown in Figure 1. This system assumes partial observability in that only agents on the outside of the flock near an approaching predator are able to see the attacking direction of the predator. They must use consensus to inform the rest of the flock about the attacking direction of the predator, which is then used with reinforcement learning to learn a target (a safe place) to move toward. That target is then used by a flocking algorithm to give each agent a control input to move each agent toward the target in a flocking formation.

The contributions of this article are then as follows:

- Utilization of consensus between agents for state approximation in reinforcement learning.
- Cooperative learning with a large number of agents.
- Implementation of function approximation to reduce state space for a large number of agents used.
- Integration of reinforcement learning and flocking to learn where to flock.
- A fast and accurate consensus algorithm for a large number of agents.
- An entirely distributed system.

### Paper organization

The organization of the remainder of this article is as follows. In “Flocking control” section, a method for flocking is introduced. The “Multiagent learning” section goes over the multiagent learning that used to ensure the agents flock together to the same target. The “Consensus for multiagent state approximation” section details a method of state approximation called consensus for the agents. This is followed by “Simulation and results” section, which details the combination of flocking, reinforcement learning, and consensus into a hybrid system for the agents to learn to flock away from a predator. Lastly, “Conclusion and future work” section covers the conclusion with analysis and potential future development.

## Flocking control

### Flocking control algorithm

In this section, the flocking algorithm used for the hybrid system is presented. To learn to avoid predators, the agents must be able to flock together. Using flocking methodologies presented in the literature,^33^ a network topology consisting of a graph *G* that is a pair (*V*, *E*) with a set of vertices *V* = {1, 2, . . . , *n*} and edges E⊆{(i,j):i,j∈V,j≠i}. In this graph, the agents are considered vertices, and the edges are communication links between neighboring agents. Using agents modeled as particles, the equations of motion are given by

(1) $$\{\begin{array}{l} {\dot{q}}_{i}=p_{i}\\ {\dot{p}}_{i}=u_{i} \end{array}$$

where **q**_*i*_ is the position of agent *i*, **p**_*i*_ is the velocity, and **u**_*i*_ is the acceleration or the control input.

The neighbors of an agent can be determined by

(2) $$N_{i}=\left\{j\in V:\left\|q_{j}-q_{i}\right\|<r\right\}$$

where ||.|| is the Euclidean norm and *r* is the interaction range of an agent.

There are many formations that flocking can take, but the formation used here is an *α*-lattice formation in which

(3) $$\left\|q_{j}-q_{i}\right\|=d\;\forall j\in N_{i}(q)$$

for desired distance *d*, where *d* = *r*/*k* for a scale factor *k*.

In flocking, each agent determines its control input with a gradient-based term $f_{i}^{g}$ given by^33^

(4) $$f_{i}^{g}=c_{1}\sum_{j\in N_{i}}\phi_{\alpha }\left({\left\|p_{j}-p_{i}\right\|}_{\sigma }\right)n_{ij})$$

where *c*_1_ is a positive constant, $n_{ij}=\sigma_{\varepsilon }\left(q_{j}-q_{i}\right)=\left(q_{j}-q_{i}\right)/\sqrt{1+\varepsilon {\left\|q_{j}-q_{i}\right\|}^{2}}$, and $\phi_{\alpha }(.)$ is a pairwise attractive/repulsive force to maintain the desired distance *d* between agents. With σ-norm, $\|\cdot {\|}_{\sigma }$ is given by $\|x{\|}_{\sigma }=1/\varepsilon \left[\sqrt{1+\varepsilon \|x{\|}^{2}}-1\right]$ that is differentiable every-where for ε>0. An obstacle avoidance term is given by $f_{i}^{o}$ that is the repulsive force of $f_{i}^{g}$ using points on obstacles as virtual neighbors $N_{i}^{\beta }$ given by

(5) $$f_{i}^{o}=\sum_{j\in N_{i}^{\beta }}b_{ij}(q)\left(p_{j}-p_{i}\right)$$

where $b_{ij}(q)=p_{h}\left({\left\|q_{j}-q_{i}\right\|}_{\sigma }/\|r{\|}_{\sigma }\right)$ is an element at row *i* and column *j* of an adjacency matrix over the interval [0, 1) for virtual neighbors *j*. A velocity consensus term $f_{i}^{d}$ is given by

(6) $$f_{i}^{d}=c_{2}\sum_{j\in N_{i}}a_{ij}(q)\left(p_{j}-p_{i}\right)$$

where $a_{ij}(q)=p_{h}\left({\left\|q_{j}-q_{i}\right\|}_{\sigma }/\|r{\|}_{\sigma }\right)$ is an adjacency matrix over the interval [0,1), and *c*_2_ is a positive constant. $p_{h}(.)$ is a bump function that smoothly varies between 0 and 1. One possible definition is given by

(7) $$p_{h}(z)=\{\begin{array}{ll} 1 & z\in [0,h)\\ \frac{1}{2}\left[1+\cos\left(\pi \frac{\left(z-h\right)}{\left(1-h\right)}\right)\right] & z\in [h,1]\\ 0 & \text{ otherwise } \end{array}$$

where h∈(0,1). A navigational term, $f_{i}^{\gamma }$, that determines the direction the agents should be moving toward, is given by

(8) $$f_{i}^{\gamma }=-c_{1t}\left(q_{i}-q_{t}\right)-c_{2t}\left(p_{i}-p_{t}\right)$$

where *c*_1*t*_ and *c*_2*t*_ are positive constants, and **q**_*t*_ and **p**_*t*_ are the position and velocity of the target, respectively. These equations can be combined to find the control input for each agent **u**_*i*_ given by

(9) $$u_{i}=f_{i}^{g}+f_{i}^{d}+f_{i}^{\gamma }+f_{i}^{o}$$

This method allows the agents to flock together in an *α*-lattice formation toward a target location.

### Results of flocking algorithm

**Figure 2.**
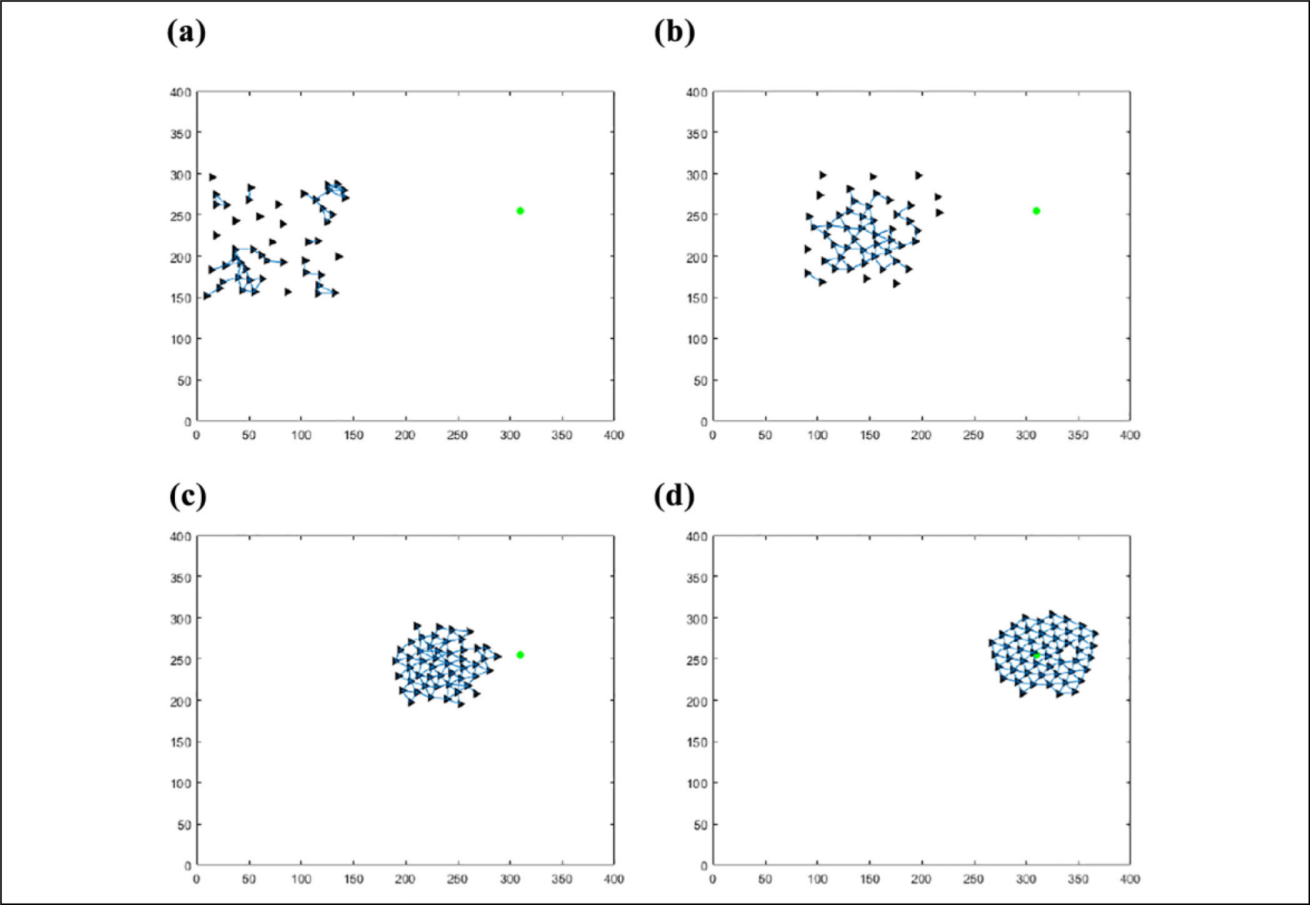
(a–d) Fifty agents flocking to the green target.

**Figure 3.**
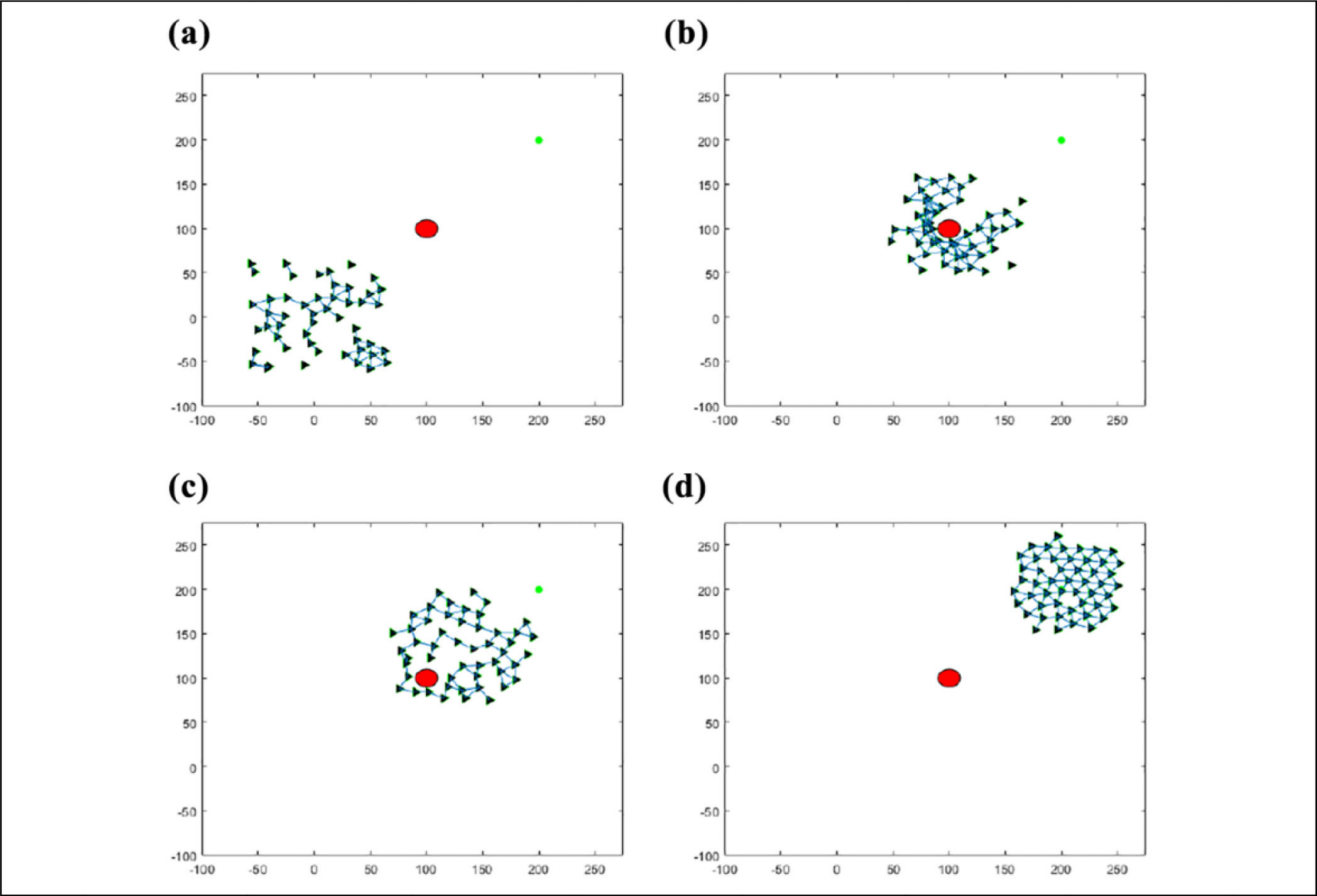
(a–d) Fifty agents flocking to the green target while avoiding the red obstacle.

The results of this flocking algorithm (**u**_*i*_) can be seen in Figure 2, where the agents are flocking to the green dot without an obstacle, and in Figure 3 with an obstacle. The agents are initialized randomly over a 120 × 120 area and flock toward the green target. The blue lines represent communication links between agents. It can be seen that the agents maintain their distance from each other without getting too far away from each other and eventually converging to an *α*-lattice formation. In the case of an obstacle, the agents manage to avoid colliding with it. This flocking algorithm thus provides a viable method of escaping from predators as well as provides a communication structure between agents to use for communications required to cooperatively learn.

## Multiagent learning

In this section, an entirely decentralized reinforcement learning method for a network to learn to flock together to specified targets is presented. Independent and cooperative learning methods are presented. In addition to this, a method of cooperative learning with function approximation is evaluated against standard cooperative learning.

### Learning model

The model of the learning algorithm is similar to that proposed in the literature.^9^ Using a state, action, and reward model for an agent *i*, let current state, action, and reward be *s*_*i*_*, a*_*i*_, and *r*_*i*_ with the next state and next action as *s′*_*i*_ and *a′*_*i*_, respectively.

#### State.

The state can be defined as *s*_*i*_ = [dir_*p*_, |*N*_*i*_|], where dir_*p*_ is the direction of a predator if detected, and |*N*_*i*_| is the number of neighbors in range for agent *i*. The state dir_*p*_ is set to 1, 2, 3, 4, 5, 6, 7, or 8 for the directions east, northeast, north, northwest, west, southwest, south, and southeast, respectively. The directions can further be divided into a larger space or smaller space if desired. In the case of multiple predators, this state space can be expanded by adding a dir_*p*_ state for each predator.

#### Action.

For actions, the agents want to move in one of eight cardinal directions to escape a predator depending on the direction the predator is coming from. These actions can be encoded as 1, 2, 3, 4, 5, 6, 7, and 8 mirroring the possible directions in the state defined above. The action list can then be defined as *A*_*i*_ = [1, 2, 3, 4, 5, 6, 7, 8]. These actions interact with the flocking algorithm in that the actions are targets in the respective direction that the agents then flock toward if chosen. If no predator is detected, the agents perform no action and stay where they are. The actions are represented as targets that an agent can choose to flock toward.

#### Reward.

The flocking algorithm used provides flocking in an *α*-lattice formation. This formation ensures that agents on the inside of the formation have up to six neighbors while agents on the outside have one to five neighbors. To match this formation, the reward is then defined as

(10) $$r_{i}=\{\begin{array}{cc} \left|N_{i}^{a}\right|\cdot D_{r} & \left|N_{i}\right|<6\\ 6\cdot D_{r} & \text{ otherwise } \end{array}$$

so that the max reward that an agent can get is 6 if it has all six neighbors to encourage flocking.

**Figure 4.**
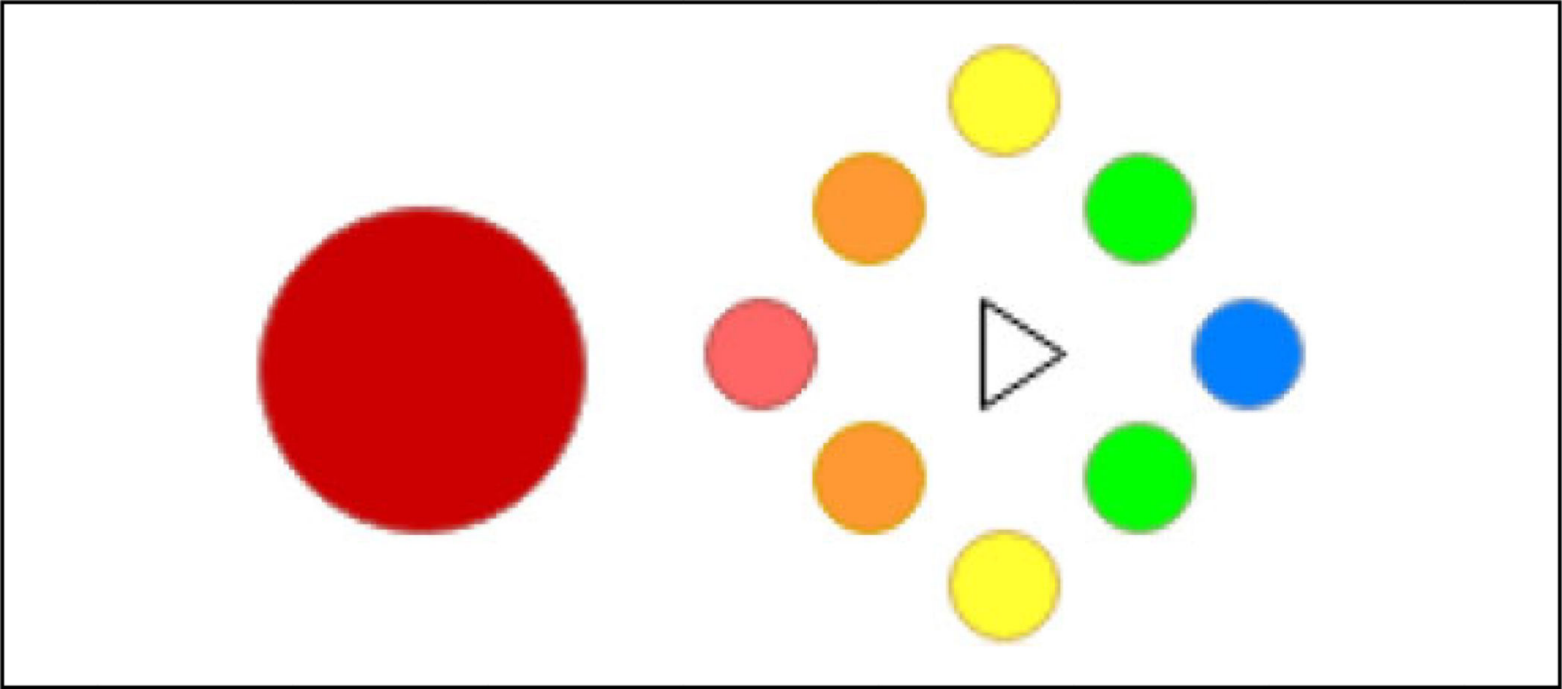
Visualization of the direction classifications. The large red circle represents the predator while the triangle represents the agent. The blue circle represents the best target, green circles are good targets, yellow circles are average targets, orange circles are bad targets, and the small red circle is the worst target in this scenario.

The reward is then scaled depending on the direction of the predator. The scaling factor is split into five categories consisting of the best target, good targets, average targets, bad targets, and the worst target, which can be visualized in Figure 4. Agents choosing the action corresponding to the best target have their reward equal to the reward defined in equation (10). *D*_*r*_ is a scale factor that is determined as follows: Actions corresponding to good targets are scaled down to 75% of the reward above, average targets to 50%, bad targets to 25%, and the worst target to 0%. This is done to encourage the agents to learn the optimal target to go toward while maintaining the importance of flocking together. The addition of more predators multiplicatively scales the reward. For example, if there are two predators and an agent chooses an action that is a good direction for both of them, the reward will be scaled down by 75% twice. This would fail if there are eight predators with one in each direction, but in that case, there is no safe space for the agents to go.

### Cooperative learning

To learn to flock to the same target together, a cooperative learning method is implemented. Agents learning independently in this environment will take many learning episodes to converge or never converge at all which can be seen in the literature.^9^ However, to cooperatively learn, each agent must first do independent learning^47^ for an individual table, *Q*_*i*_, as follows:

(11) $$Q_{i}^{k+1}\left(s_{i},a_{i}\right)\leftarrow Q_{i}^{k}\left(s_{i},a_{i}\right)+\alpha [r_{i}^{k}+\gamma \;\max_{{a^{\prime }}_{i}\in A_{i}}Q_{i}^{k}\left(s_{i}^{\prime },a_{i}^{\prime }\right)-Q_{i}^{k}\left(s_{i},a_{i}\right)]$$

where *α* is a learning rate and γ is a discounting factor. This independent learning is not capable of converging in any reasonable amount of time for this application, so cooperative learning must be used. After performing independent learning, the Q-table of each agent is further updated by communicating with its neighbors using the following^9^:

(12) $$Q_{i}^{k+1}\left(s_{i},a_{i}\right)\leftarrow wQ_{i}^{k}\left(s_{i},a_{i}\right)+(1-w)\frac{\sum_{j=1}^{\left|N_{i}\right|}Q_{j}^{k}\left(s_{j},a_{i}\right)}{\left|N_{i}\right|}$$

where *w* is a weight, such that 0 ≤ *w* ≤ 1 to determine how much an agent should trust neighbors versus itself. It can be seen *w* = 1 would mean the agent only trusts itself, and *w* = 0 would mean the agent only trusts its neighbors. The weight chosen can either be a static value or in this application, the weight is defined as $w=\frac{1}{\left|N_{i}\right|+1}$ so that each agent equally trusts each other agent. Dividing the sum by |*N*_*i*_| is required so that over the course of the learning, the Q-values do not converge to infinity too quickly. Note that the update from the neighbors is based on the neighbors state *s*_*j*_ and the agents own action *a*_*i*_.

### Action selection

The action selection of an agent is based on the maximum Q-value approach^48,58^ in which the action with the highest Q-value for a given state is the action chosen. This method of choosing the action is highly exploitative with no exploration. To introduce exploration, we use *ε*-greedy.^47^ We use a small probability 0 ≤ *ε*_*g*_ ≤ 1 in which to ignore the highest Q-value and instead select an action at random. This can be modeled as follows

(13) $$a_{i}=\{\begin{array}{cc} a_{\max}\in A_{i} & \varepsilon_{g}<\text{ random }(0,\ldots ,1)\\ a_{\text{random }}\in A_{i} & \text{ otherwise } \end{array}$$

This random action selection allows an agent to explore a new action that might return a higher reward. The same action selection can be used for function approximation learning replacing the Q-value with the ***θ*** parameter vector.

### Function approximation

Despite the cooperative *Q* learning algorithm performing better than independent learning, as seen in the literature,^9^ it still can be improved upon to get better results and faster convergence. The direction of the predator dir_*p*_ is already discretized into eight directions, however, the number of neighbors |*N*_*i*_| grows in size with the number of agents used. Due to the random initialization of agents at the start of each episode, it is possible for each agent to be a neighbor of each other agent. However, as the episode progresses and the *α*-lattice formation is achieved, this state will have one of seven values for either no neighbor or one to six neighbors. This state size is not particularly large, but the *Q* values for higher neighbor amounts are ideally found to ensure smooth flocking to the target. A radial basis function (RBF) method of function approximation is used to achieve quicker learning. A fixed sparse representation method was explored, but RBF was found to perform better. The state, action, and reward representations remain the same, but the number of neighbors |*N*_*i*_| is now being approximated using RBF.

### Radial basis function.

Function approximation allows to approximate a state space rather than just discretize it. There are many methods of doing function approximation, but the method that seemed most applicable was a simple RBF approach. The RBF scheme maps the original *Q* table to a parameter vector ***θ*** as^49,50^

(14) $$Q_{i}\left(s_{i},a_{i}\right)=\sum \phi_{l}\left(s_{i},a_{i}\right)\theta_{i,l}=\phi^{T}\left(s_{i},a_{i}\right)\theta_{i}$$

where the RBF kernel *ϕ* is a column vector of length *l* · |{*A*}|. The output of the *l*′ th RBF kernel is given as

(15) $$\phi_{l}(s)=e^{-\frac{{\left\|s-{\bar{s}}_{l}\right\|}^{2}}{2\mu_{l}^{2}}}$$

where *s* is the current state, ${\bar{s}}_{l}$ is the center of the RBF kernel *l*, and *μ*_*l*_ is the radius of the RBF kernel *l* producing the shape of a Gaussian bell. A larger *μ*_*l*_ thus produces a flatter RBF.

### Function approximation learning.

The cooperative learning algorithm from equations (11) and (12) is still used to learn, however, it is modified to account for the parameter vector ***θ*** and RBF kernel ϕ in equation (15) rather than *Q*-values.^49^ The independent part of RBF learning is then given as

(16) $$\begin{array}{l} \theta_{i}^{k+1}\leftarrow \theta_{i}^{k}+\alpha \left[r_{i}^{k}+\gamma \;\max_{a^{\prime }\in A_{i}}(\phi^{T}\left(s_{i}^{\prime },a_{i}^{\prime }\right)\theta_{i}^{k}\right)\\ -\left(\phi^{T}\left(s_{i},a_{i}\right)\theta_{i}^{k}\right]\phi \left(s_{i},a_{i}\right) \end{array}$$

with the same learning rate *α* and discount factor *γ* as before. The cooperative portion is as follows

(17) $$\theta_{i}^{k+1}=w\theta_{i}^{k}+(1-w)\frac{\sum_{j=1}^{\left|N_{i}\right|}\theta_{j}^{k}}{\left|N_{i}\right|}$$

The key difference here is that each agent must now communicate a ***θ*** vector rather than just a single *Q* table value since the entire ***θ*** vector approximates the state. In standard reinforcement learning, the learning is conducted over multiple episodes. The episodes here consist of iterations of agents flocking toward the targets corresponding to their actions. Learning is concluded when the agents all learn the same action for each given state. After learning all states, the agents will have a learned ***θ*** table that can be used to guide the agents in a flock to safe locations away from predators. The algorithm for this learning is then given in Algorithm 1.

### Comparison of learning algorithms

**Figure 5.**
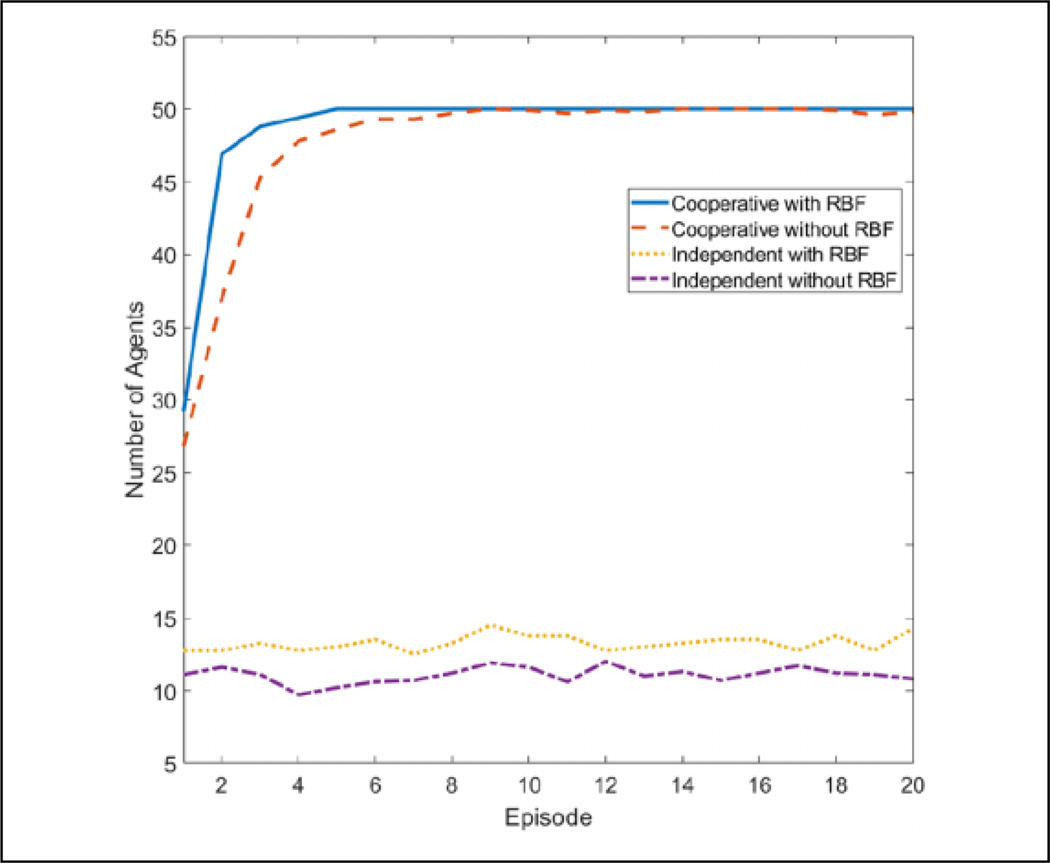
Comparison of convergence between RBF and *Q* learning with and without cooperative learning. All four algorithms were run 10 times for 20 episodes, and the results were averaged. Over the course of these runs, cooperative RBF was able to converge within four episodes while cooperative *Q* learning was unsuccessful in fully converging within 20 episodes. Both independent algorithms were not able to learn. RBF: radial basis function.

If we use 50 agents *n* = 50 with eight discrete directions |dir_*p*_| = 8 and eight actions corresponding to those directions |{*A*}| = 8, the *Q* table would be of size 50 × 8 · × 50 × 8 = 1.6 × 10^5^. Since the directions are already discretized and the number of actions and agents cannot be reduced, only the neighbor dimension |*N*_*i*_| can be reduced. For this application, eight RBF kernels were used to approximate the space although less or more will probably perform similarly. The ***θ*** table is then of size 50 × 8 · 8 × 8 = 2.56 × 10^4^, which is approximately one-sixth of the original size. In a state space in which the direction of predators is ignored |dir_*p*_| = 1, the *Q* size is 2 × 10^4^ while ***θ*** is 3.2 ×10^3^. The results of this learning in this state space are shown in Figure 5. It is clear that using cooperative learning with function approximation, the performance is significantly better than without in both space and training time required. Because of this large gap in learning effectiveness, only cooperative learning with function approximation is used for the larger state space, where |dir_*p*_|= 8.

**Algorithm 1.** Function approximated distributed cooperative learning.

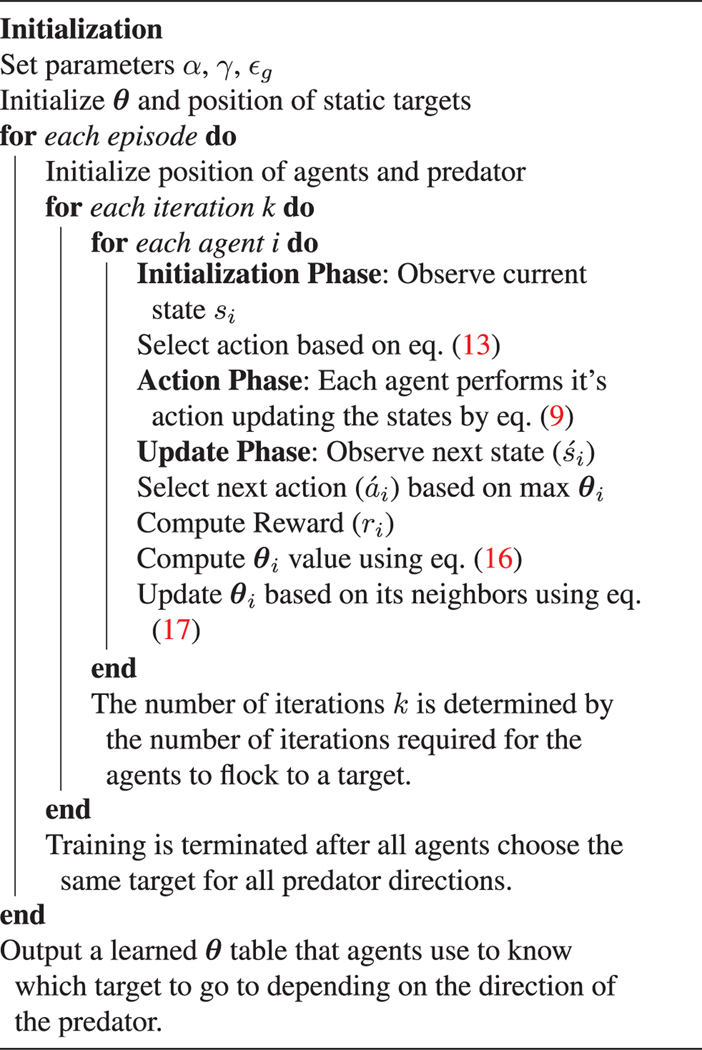

## Consensus for multiagent state approximation

In this section, a way of sensing and communicating the direction of a predator is presented. Each agent has a predator sensing radius *r*_*p*_ that allows them to sense a predator. If a predator is within that radius, then the agent is able to know its relative angle to the predator. These angles can be used to determine the direction the predator is coming from. However, not every agent will be in range of the predator to see the direction that it is coming from and not every agent that is in range will agree on which direction the predator is coming from. To solve this, a weighted voting method is introduced for agents to share and achieve a consensus on the direction of the predator.

### Predator sensing

**Figure 6.**
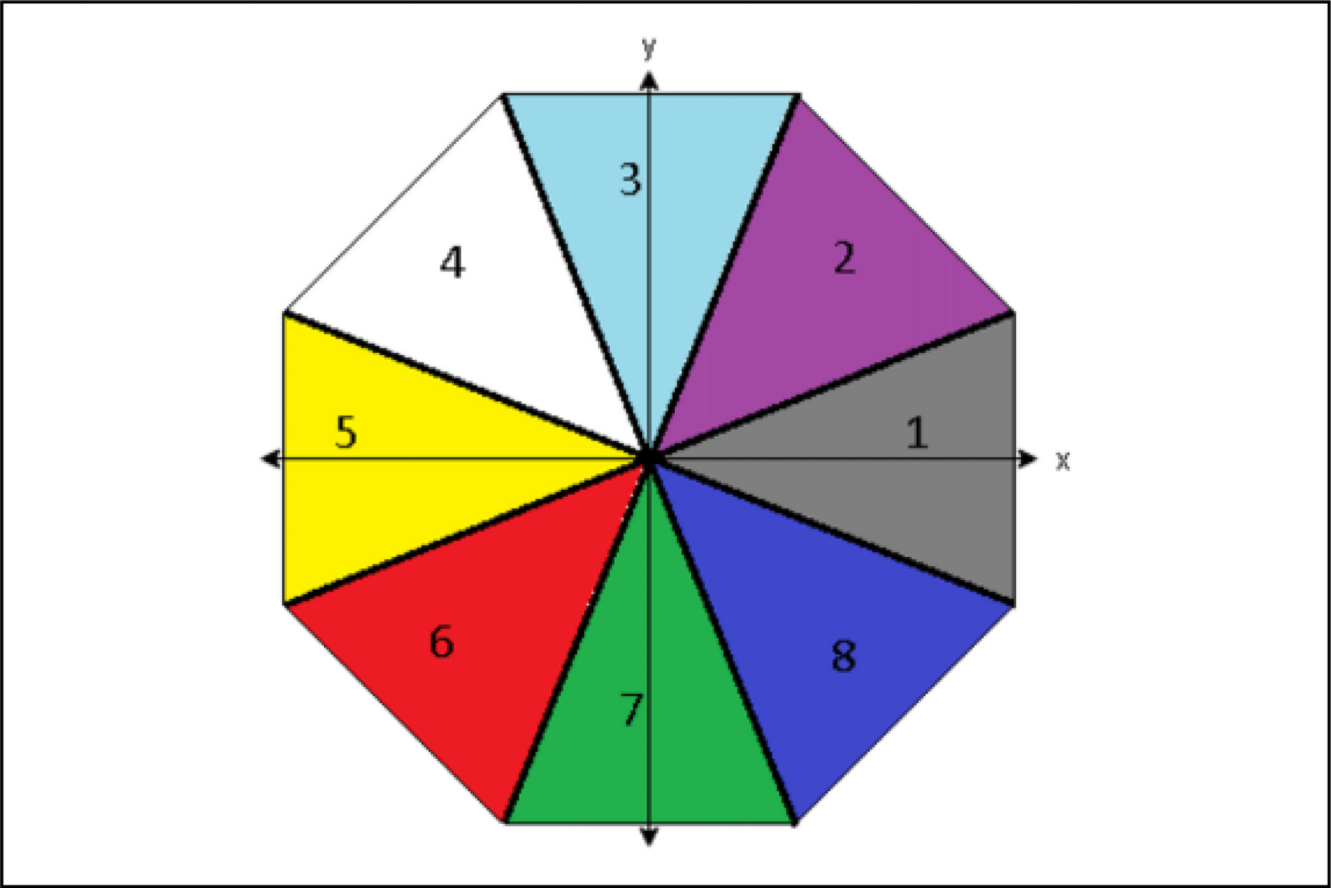
Direction and associated information values for an agent observing a predator. Each color corresponds to an information value 1 through 8 with 1 representing east, 3 representing north, and so forth.

A method of agents achieving consensus proposed in the literature^56^ is used as a start point for the weighted voting procedure. The algorithm is split into two components: a measurement step and a consensus step. For the measurement step, if an agent is in range of the predator, then it performs a measurement of the relative direction of the predator. As mentioned previously, the direction is discretized into eight evenly split directions. These directions are assigned to an information vector info_*i*_ as

(18) $${\text{ info }}_{i}=\{\begin{array}{ll} 1 & 0\leq w_{p}<22.5,337.5\leq w_{p}\leq 360\\ 2 & 22.5\leq w_{p}<67.5\\ 3 & 67.5\leq w_{p}<112.5\\ 4 & 112.5\leq w_{p}<157.5\\ 5 & 157.5\leq w_{p}<202.5\\ 6 & 202.5\leq w_{p}<247.5\\ 7 & 247.5\leq w_{p}<292.5\\ 8 & 292.5\leq w_{p}<337.5 \end{array}$$

where *w*_*p*_ is the angle between the agent and the predator such that 0 ≤ *w*_*p*_ ≤ 360. These directions are visualized in Figure 6. These directions do not have to be symmetrical or positioned, as have been positioned here. There can also be more or less directions as desired. However, the directions for this application were chosen as eight evenly divided directions such that they are all 45° in width, 360/8 = 45, and they are aligned to the cardinal directions.

Each agent’s information vector is assigned a weight or a belief factor weight_*i,d*_. This weight vector is of size number of agents by the number of directions *n* · |dir_*p*_| and is determined from an agent’s measurement given by equation (19), where *w*_*m*_ is the middle angle of the direction measured, info_*i*_ + 1 is the next direction counterclockwise, and info_*i*_ –1 is the next direction clockwise. For example, if info_*i*_ = 1, then info_*i*_ − 1 = 8. The scale factor $\frac{w_{m}-w_{p}+45}{45}$ splits the distance weight $1-\frac{\left\|q_{p}-q_{i}\right\|}{r_{p}}$ into two directions of the weight vector. This scale factor is between 0 and 1, and is determined by where the measured angle is relative to the center of the direction. For example, the center of direction one *w*_*m*_ is 0°, and if the measured angle *w*_*p*_ is 0°, then $\frac{w_{m}-w_{p}+45}{45}=1$. Thus, the weight_*i*,1_ = $\left(1-\frac{\left\|q_{p}-q_{i}\right\|}{r_{p}}\right)$ · 1. If the measured angle *w*_*p*_ was 22.5, then $\frac{w_{m}-w_{p}+45}{45}=0.5$ and $1-\frac{w_{m}-w_{p}+45}{45}=0.5$. Thus, weight_*i*,1_ = weight_*i*,2_ = $1-\frac{\left\|q_{p}-q_{i}\right\|}{r_{p}})*0.5$. The idea here is to assign weight based on closeness to the predator and closeness to the center of the directions. Once the information and weight has been found for all agents, we can then run a consensus based on weighted voting

(19) $${\text{weight}}_{i,d}=\{\begin{array}{ll} \left(1-\frac{\left\|q_{p}-q_{i}\right\|}{r_{p}}\right)*\left(\frac{w_{m}-w_{p}+45}{45}\right) & \|q_{p}-q_{i}\|<r_{p},d=\inf o_{i}\\ \left(1-\frac{\left\|q_{p}-q_{i}\right\|}{r_{p}}\right)*\left(1-\frac{w_{m}-w_{p}+45}{45}\right) & \|q_{p}-q_{i}\|<r_{p},d=\inf o_{i}+1,w_{p}>w_{m}\\ \left(1-\frac{\left\|q_{p}-q_{i}\right\|}{r_{p}}\right)*\left(1-\frac{w_{p}-w_{m}+45}{45}\right) & |q_{p}-q_{i}\|<r_{p},d=\inf o_{i}-1,w_{p}<w_{m}\\ 0 & \text{otherwise} \end{array}$$

### Consensus

For consensus, each agent updates its information info_*i*_ and weight weight_*i,d*_ based on its neighbors *N*_*i*_. The goal is for all agents to agree on the same info_*i*_ and for that info_*i*_ to be as accurate as possible, thus achieving consensus on the direction of the predator. To do this, a weighted voting method is implemented, where the weights for an agent and its neighbors are summed together into the weighted direction vector weight_*i*_, such that ${\text{ weight }}_{i}={\text{ weight }}_{i}+\sum_{j=1}^{\left|N_{i}\right|}{\text{ weight }}_{j}$. The info_*i*_ is then set to the direction that has a maximum weight info_*i*_ = max_*d*_ (weight_*i,d*_). The weight and information are updated for all agents for a set amount of iterations *c*_*m*_ in this manner, and then, the weight for each agent is updated to the maximum weight among itself and its neighbors such that weight_*i*_ = max_weight_ (weight_*Ni*_ ⋃ weight_*i*_). Sharing the maximum weight after the set amount of iterations allows for all agents to converge to the same predator direction in a quick manner. The measurement and consensus steps can be combined, as seen in Algorithm 2.

Using this algorithm, info_*i*_ is found for each agent, and given enough iterations, the proposed consensus will converge to the same value for all agents. This value is used to determine the state dir_*p*_ in the reinforcement learning component.

### Validation

**Figure 7.**
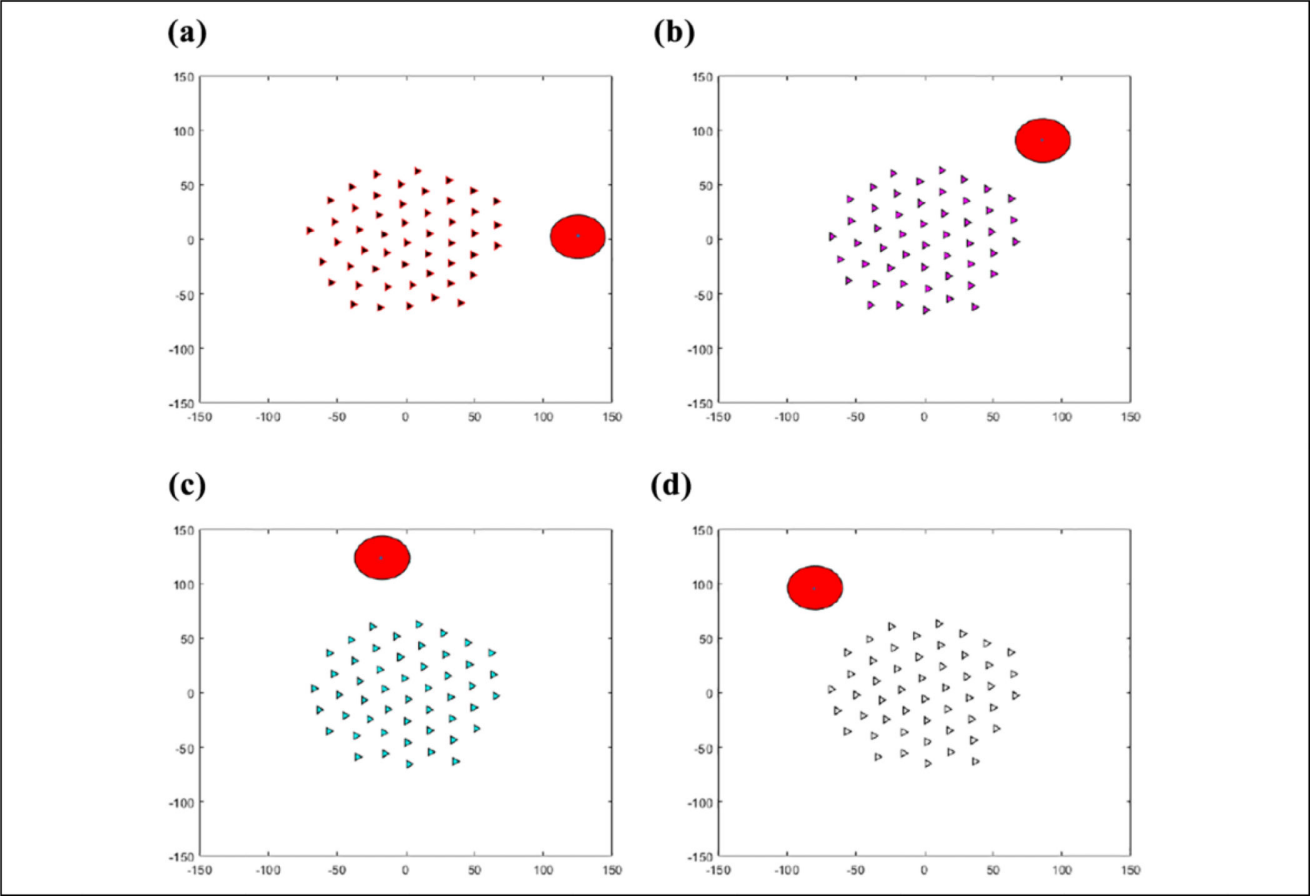
(a–d) Predator moving in circle around flock with consensus updating the state of the agents based on the direction of the predator.

**Figure 8.**
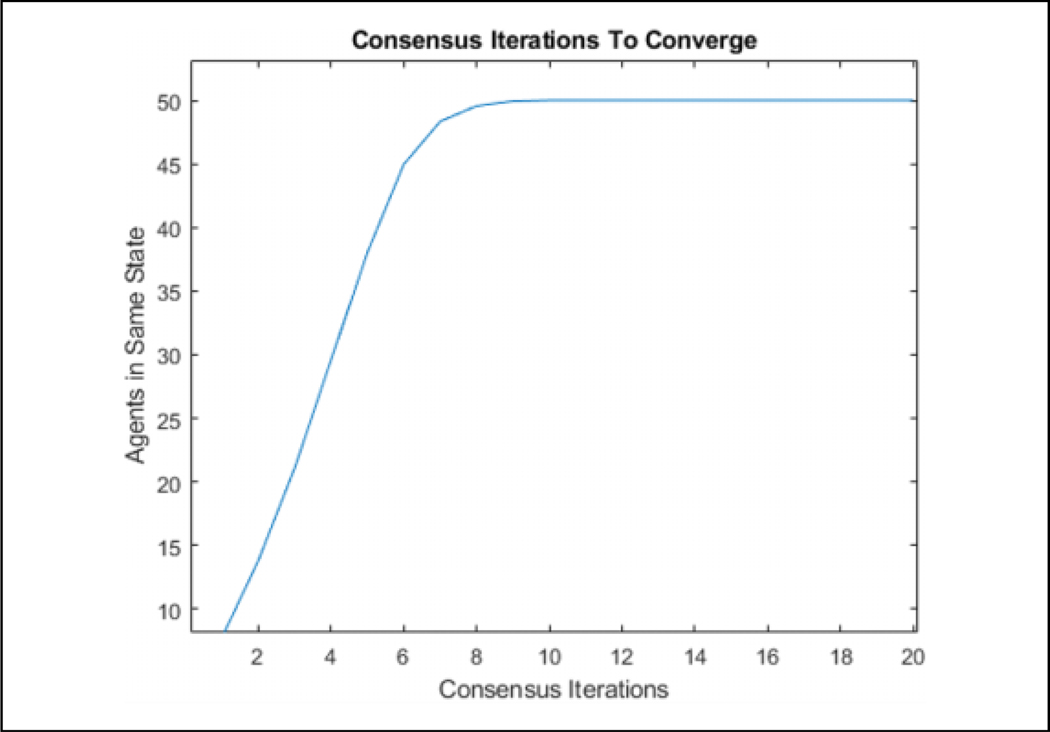
The number of consensus iterations for all agents to converge to the same state.

**Figure 9.**
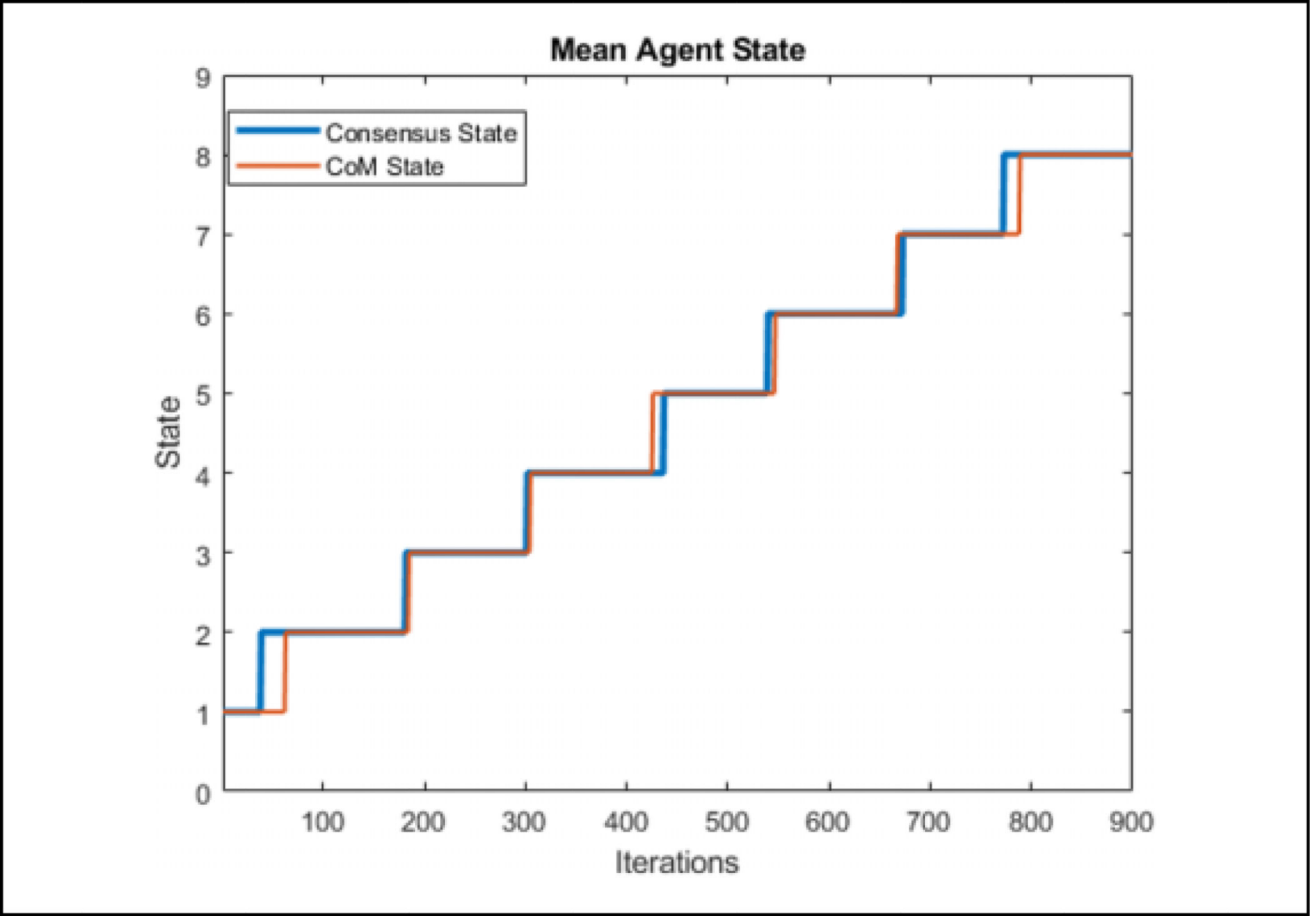
The average info_*i*_ for the agents in each iteration. Some states occur for a longer duration due to the shape of the flock.

**Figure 10.**
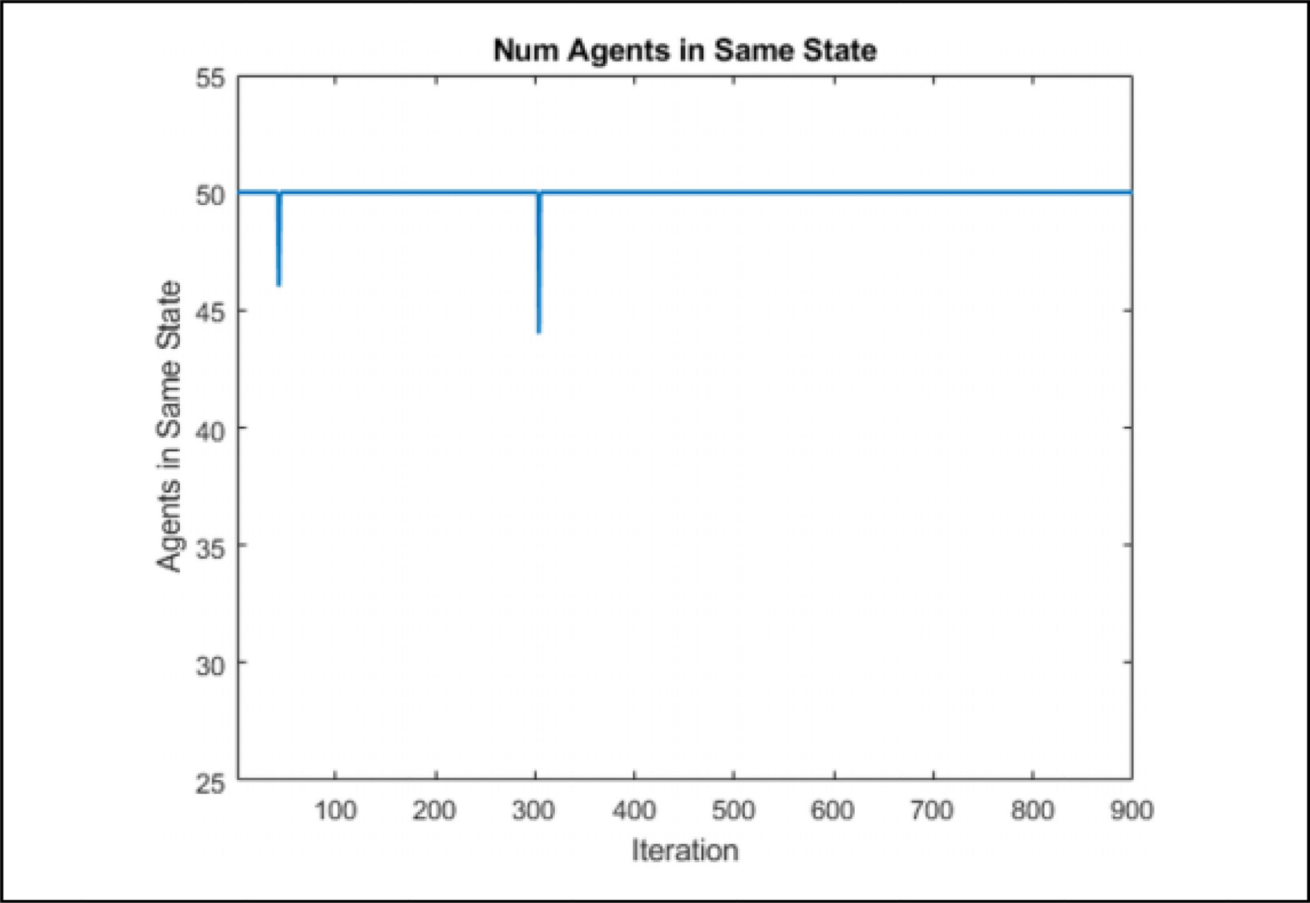
The number of agents in the same state when *c*_*m*_ is arbitrarily high. There are multiple predator positions that result in the agents not fully converging even given 40 iterations.

**Figure 11.**
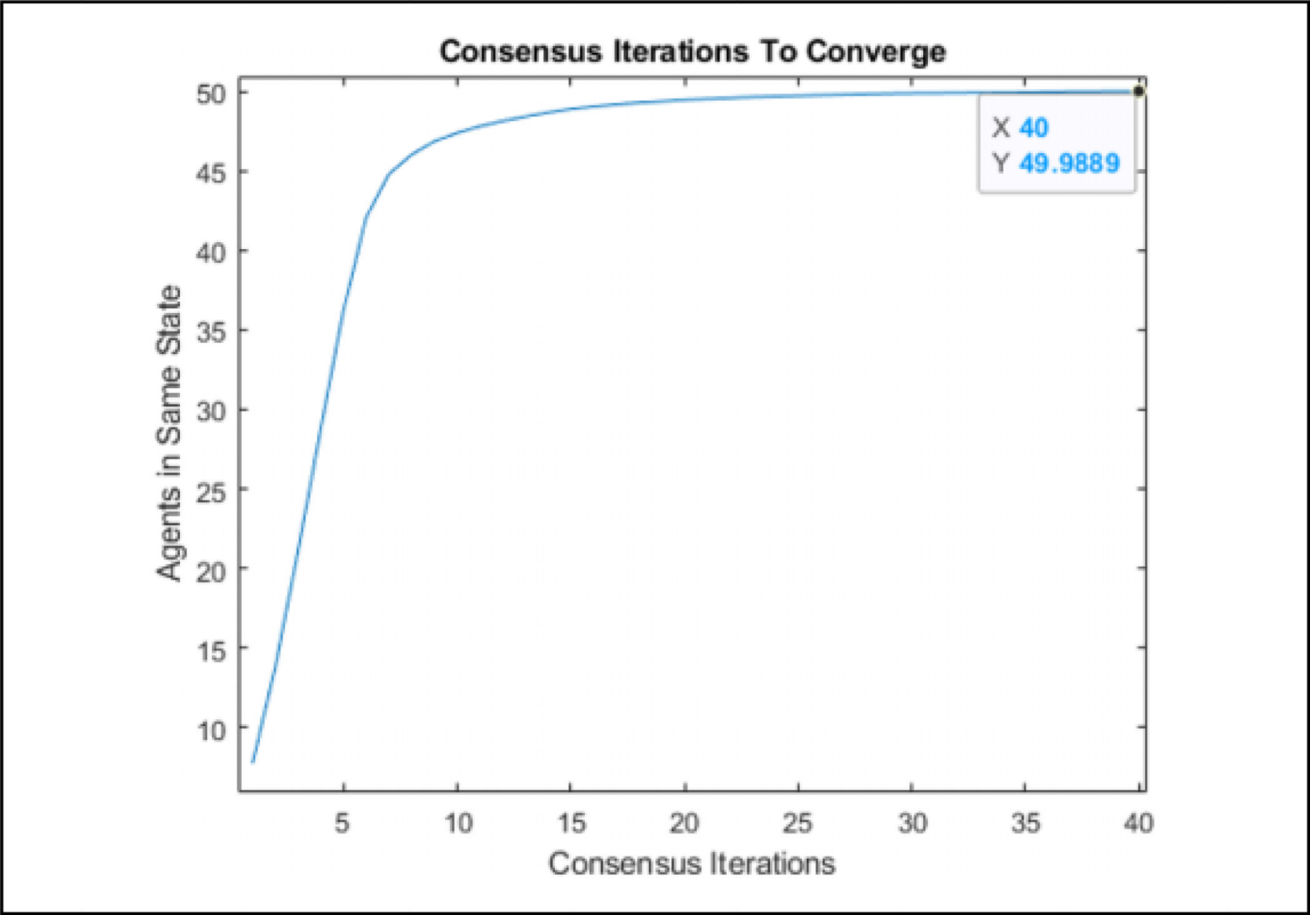
The convergence of the agents state when *c*_*m*_ is arbitrarily high. Given 40 consensus iterations, it is not able to fully converge.

Algorithm 2 is tested in an environment, where 50 agents are flocking to a static position while seeing a predator, denoted by a large red circle, moving in a circle around the flock over 900 iterations, as can be seen in Figure 7. Visually, the agents that are represented by the triangles change color in association with the direction they perceive the predator to be in after consensus. The consensus component was allowed to run for 20 consensus iterations and was found that all agents converged to the same info_*i*_ within that duration. The number 20 was arbitrarily chosen as a large number to ensure that the agents have enough iterations to achieve a consensus. One run of the average time it took to converge for each of the 900 iterations can be seen in Figure 8. Figure 9 shows the comparison of the state found through consensus to the actual state relative to the center of mass of the flock. It can be seen that it is not always perfectly accurate, but this can be attributed to a lack of full observability and lack of symmetry in the flock. It was always able to fully converge for varying values of *c*_*m*_, which can be seen in Table 1. However, if one does not do the maximum weight sharing by setting *c*_*m*_ to an arbitrarily high value, the algorithm will not fully converge even given up to 40 iterations, as can be seen in Figures 10 and 11.

**Figure 12.**
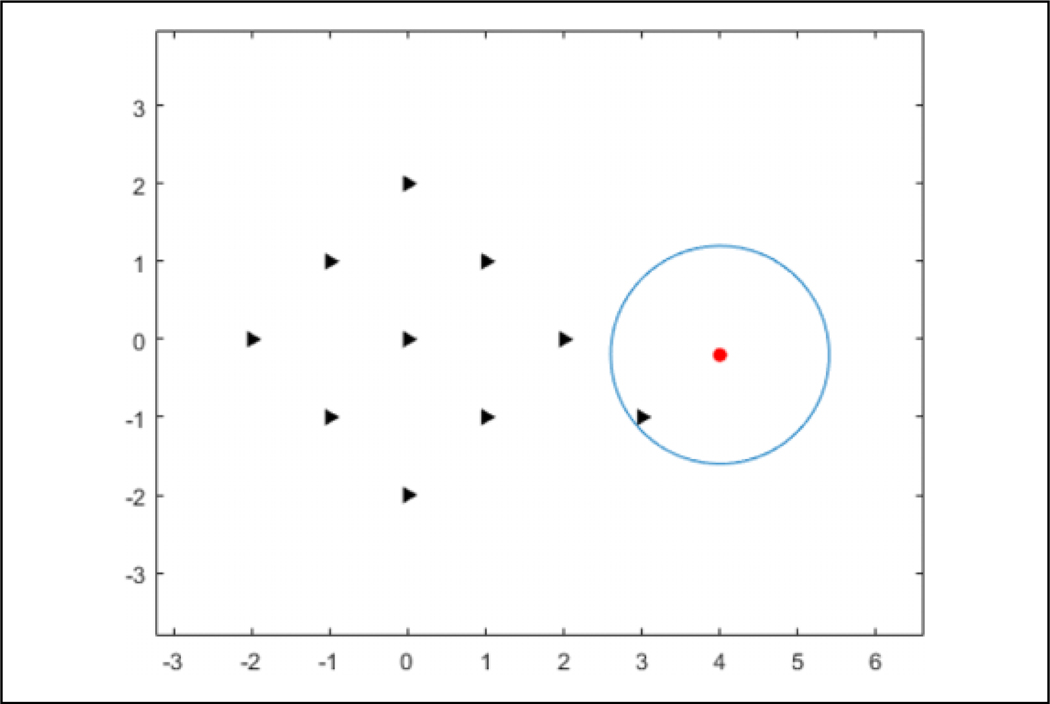
The cause of error of the consensus algorithm. Despite the predator being east of the flock as a whole the only agent in range senses it as northeast causing the error seen in Table 1.

From Table 1, we can see that if the largest measured weight is spread from one side of the flock to the other, *c*_*m*_ = 0, it takes 8.2 iterations to completely reach every agent in the flock. That amount of iterations to achieve a consensus is thus not able to be made smaller due to the network communication limitations and size of the flock. By adding additional weighted voting iterations *c*_*m*_, we can see that it takes additional iterations to converge for each weighted voting iteration added. It is also clear that adding more weighted voting iterations does not necessarily increase the accuracy of the consensus as can be seen by comparing *c*_*m*_ = 2 and *c*_*m*_ = 3. By letting *c*_*m*_ = 10, it can be seen that there is about an 1:5% increase in performance compared to *c*_*m*_ = 2 but at a much higher computational cost. The error in accuracy can be attributed to the formation of the flock, as can be seen in Figure 12. Despite the predator being east of the flock as a whole, the agents will converge to northeast due to only one agent being in range of the predator to sense it. Using this data going forward to the hybrid system, we let *c*_*m*_ = 2 and let the number of consensus iterations be 12 to allow some margin of error to account for a poor flocking structure.

**Algorithm 2.** Consensus on direction of predator.

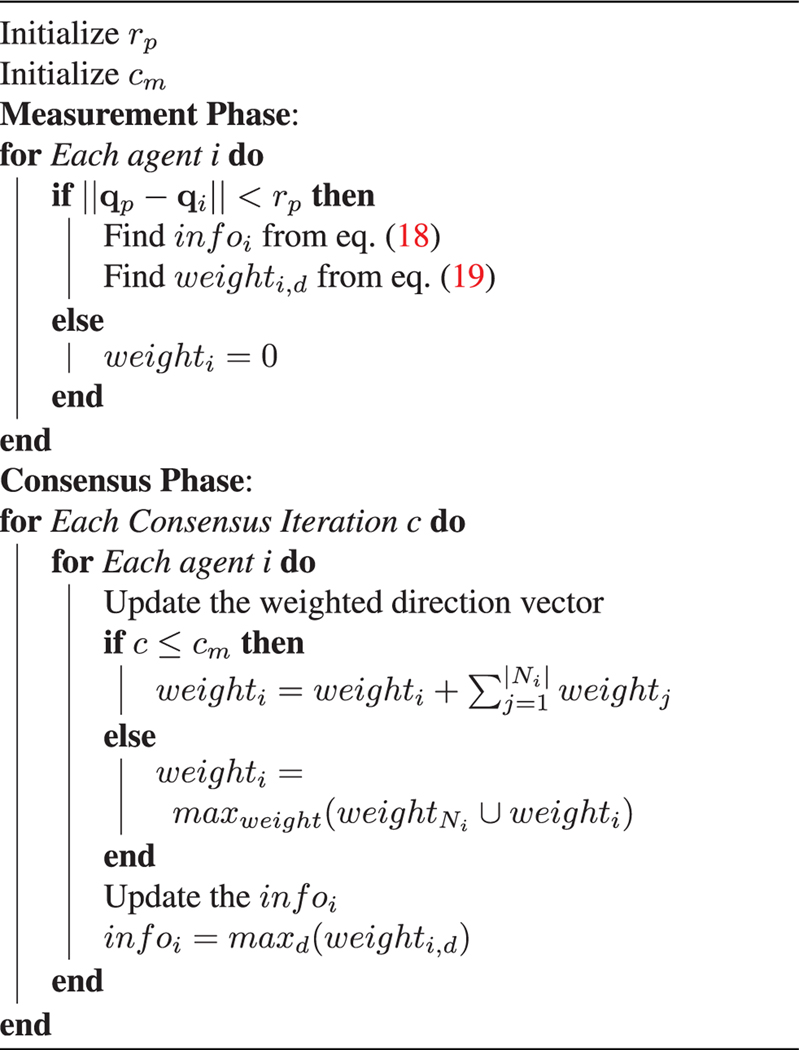

Through testing alternate consensus methods, one was able to achieve a higher accuracy. This method involved using consensus to determine the center of mass of the flock and absolute position of the predator. When each agent has that center of mass position and the position of the predator, it can then determine for itself the direction of the predator, but this approach is not used for a few reasons. First and foremost, the time it takes to reach a consensus is at least two to three times the number of iterations that the proposed approach uses, thus making it take much longer to both learn and later use practically for predator detection. Secondly, it requires that each agent has an absolute coordinate for itself and the predator rather than relative directions for those agents in range of the predator. In many environments, this may not be known or if it may have a large amount of noise associated with it, so this approach was not used.

## Simulation and results

In this section, we go over implementation details and results found for the hybrid learning system. We use consensus to determine the direction of the predator dir_*p*_. This state is then used in the multiagent learning for the ***θ*** table. The multiagent learning then produces an action, which is a target to flock toward that is used by the flocking algorithm for the agents. Finally, the flocking algorithm produces a control input for each agent to flock to its chosen target. Using this system, we can teach agents to detect and flock away from predators. The simulation was developed using MATLAB. Each learning episode consists of a certain time duration that is long enough for agents to flock to the determined target. Within each episode, the number of iterations that it is run for is determined by the time step. A smaller time step gives longer episodes but faster learning due to more communications occurring over the episode. Smaller time steps also provide for smoother flocking. Each agent is represented by plotting its position as a triangle at each time step in a two-dimensional MATLAB plot.

### Simulation environment

**Figure 13.**
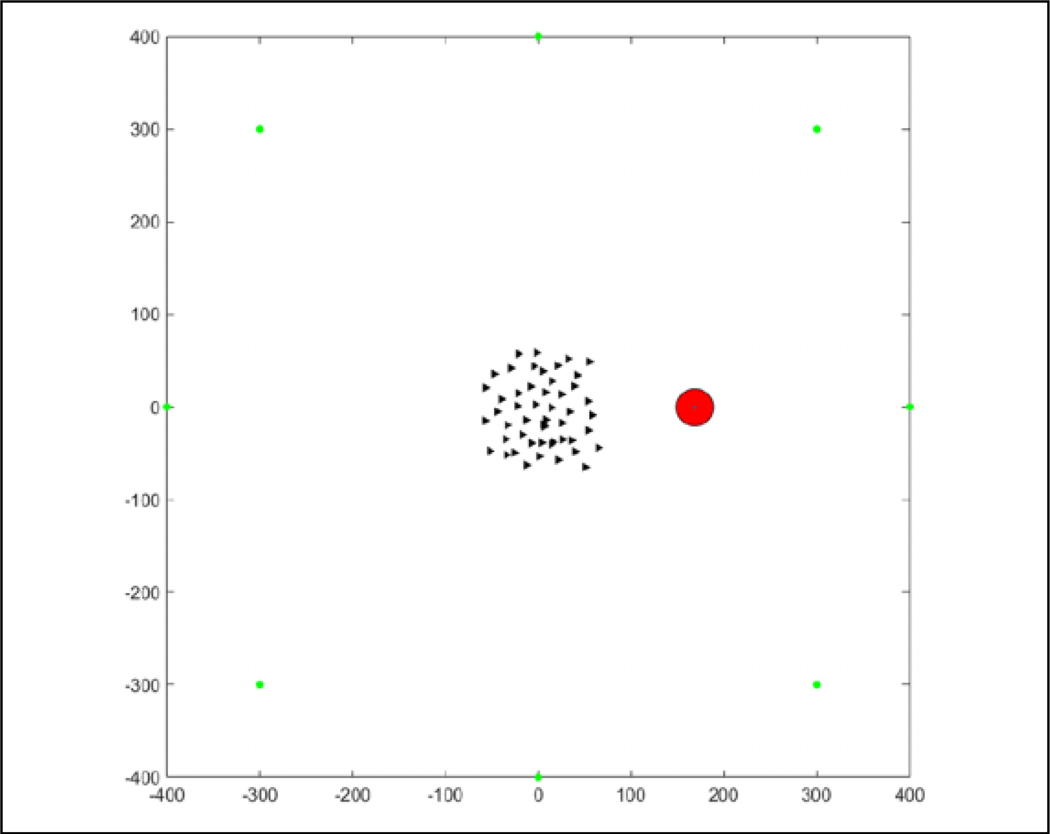
Initialization of an episode with agents randomly distributed.

The learning environment is set up in a manner, as shown in Figure 13, where the triangles represent the agents, and the large red circle represents the predator. The eight smaller green circles around the edge represent the eight static targets for the eight actions. Each episode begins by randomly initializing 50 agents in a 120 × 120 area and the predator in one of eight directions. The predator then moves toward the center of mass of the agents. The predator is placed far enough away that the agents will be fully connected to each other but not necessarily in perfect flocking formation by the time the predator gets in range. This is done to ensure that each agent is able to get the direction of the predator through consensus so that an agent does not get left behind due to being initialized too far away from the rest of the agents.

### Learning configuration.

For the single predator environment, the direction of the predator is initially east, then northeast, and so on, in a counter-clockwise rotation so that ideally every possible state dir_*p*_ is encountered once every eight episodes. The learning is conducted over 56 episodes and the results can be seen below. For the two-predator environment, the learning is conducted over 216 episodes with the reason explained below. For the *ε*-greedy action selection in equation (13), an *ε* value of 0.1 is chosen to allow the agents to explore other actions more quickly while not hindering the smoothness of flocking too extremely.

### Results

#### Single direction state.

**Figure 14.**
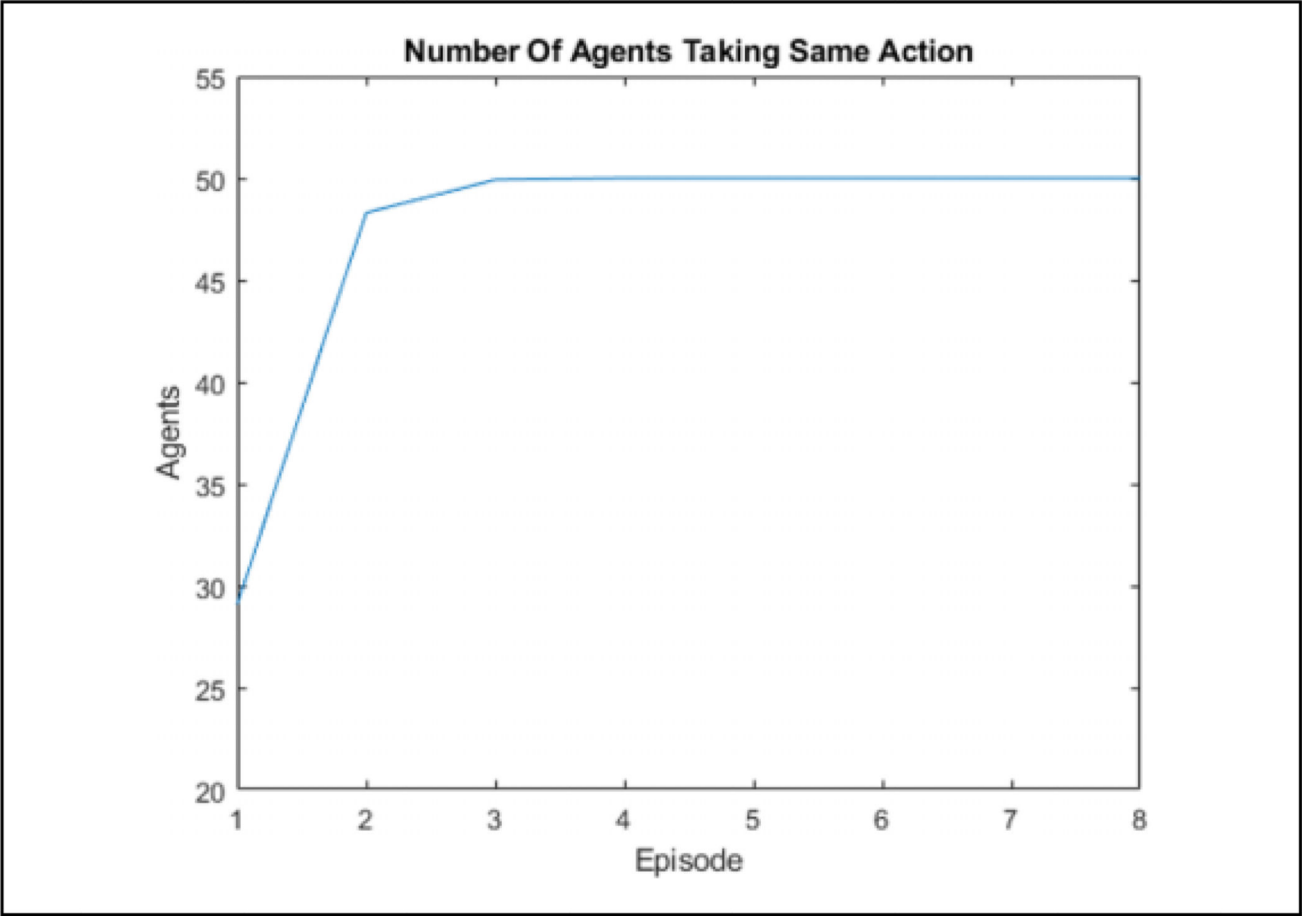
The number of agents choosing the same action for each episode for a single direction state by episode. The agents are able to fully converge by four episodes.

**Figure 15.**
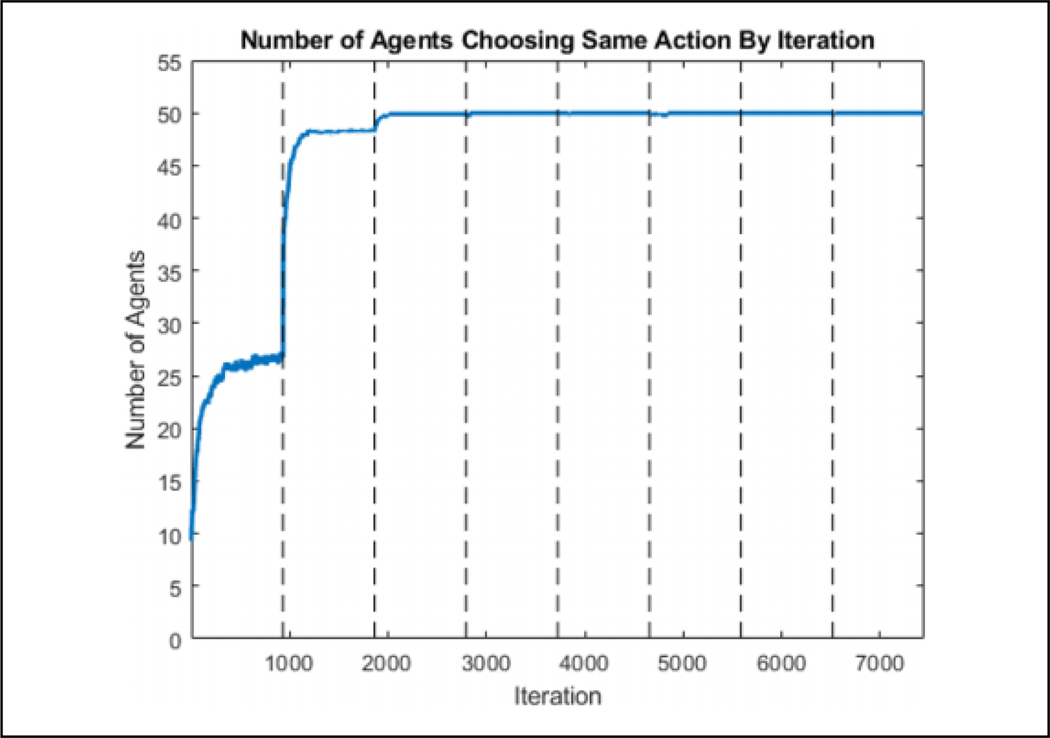
The number of agents choosing the same action for each episode for a single direction state by iteration with the dashed lines representing the start of an episode. It can be seen that most learning is done in the beginning of an episode while the flock is connected.

We first look at a scenario in which all agents are in the same direction state with the use of consensus. The average results of 10 runs over eight episodes can be seen in Figures 14 and 15. We can see that the agents are able to fully converge for a single direction state in four episodes. Thus, the theoretical number of episodes required to learn is 8 directions times 4 episodes required or 32. However, due to the nature of flocks not being perfect, it is possible for each direction to not be seen four times within those 32 episodes, so 56 episodes are used to learn for the single predator and 216 for the two-predator environment to account for slower learning due to consensus.

#### Single predator.

**Figure 16.**
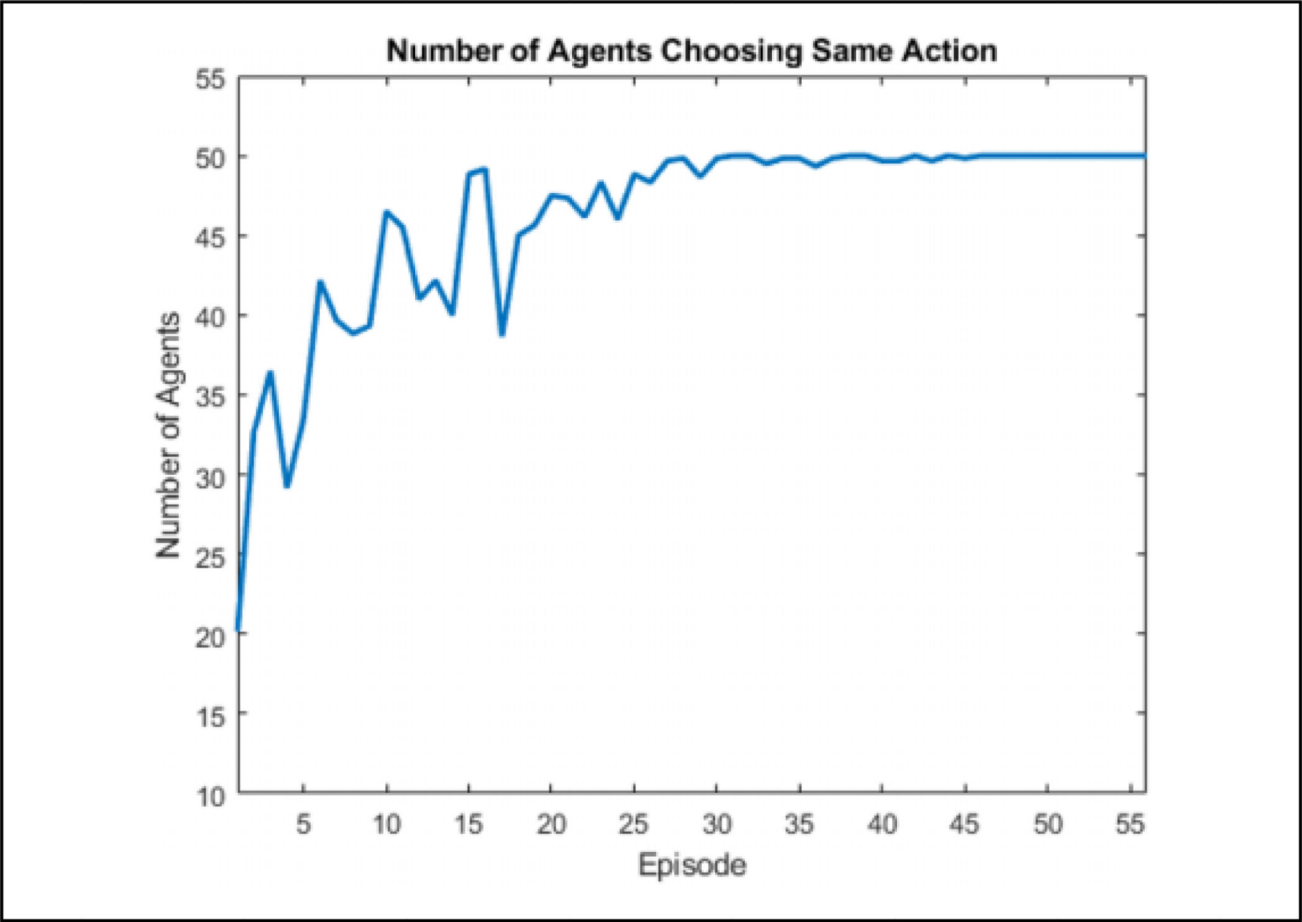
The number of agents choosing the same action for each episode for a single predator environment averaged over six runs. It can be seen that the agents converge by each direction being encountered six times.

Six runs were conducted and averaged for the single predator environment. In Figure 16, it can be seen that by episode 32, or the predator coming from each direction four times, the agents have mostly converged to the same target, but there are a few cases in which it is not fully converged until approximately episode 48 or each direction occurring six times, which is expected due to potential consensus inaccuracies.

**Figure 17.**
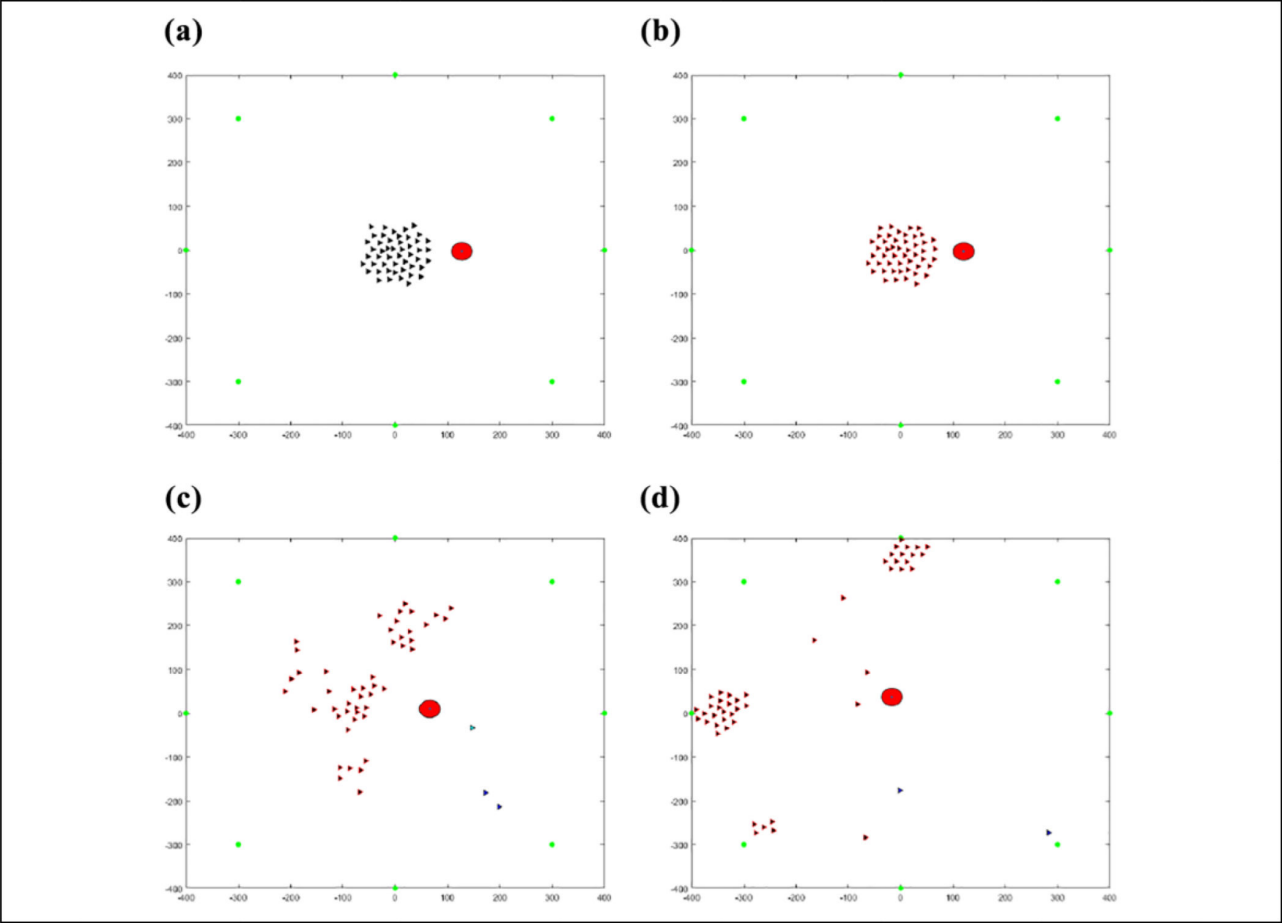
(a–d) Fifty agents flocking away from one predator before learning the same target.

**Figure 18.**
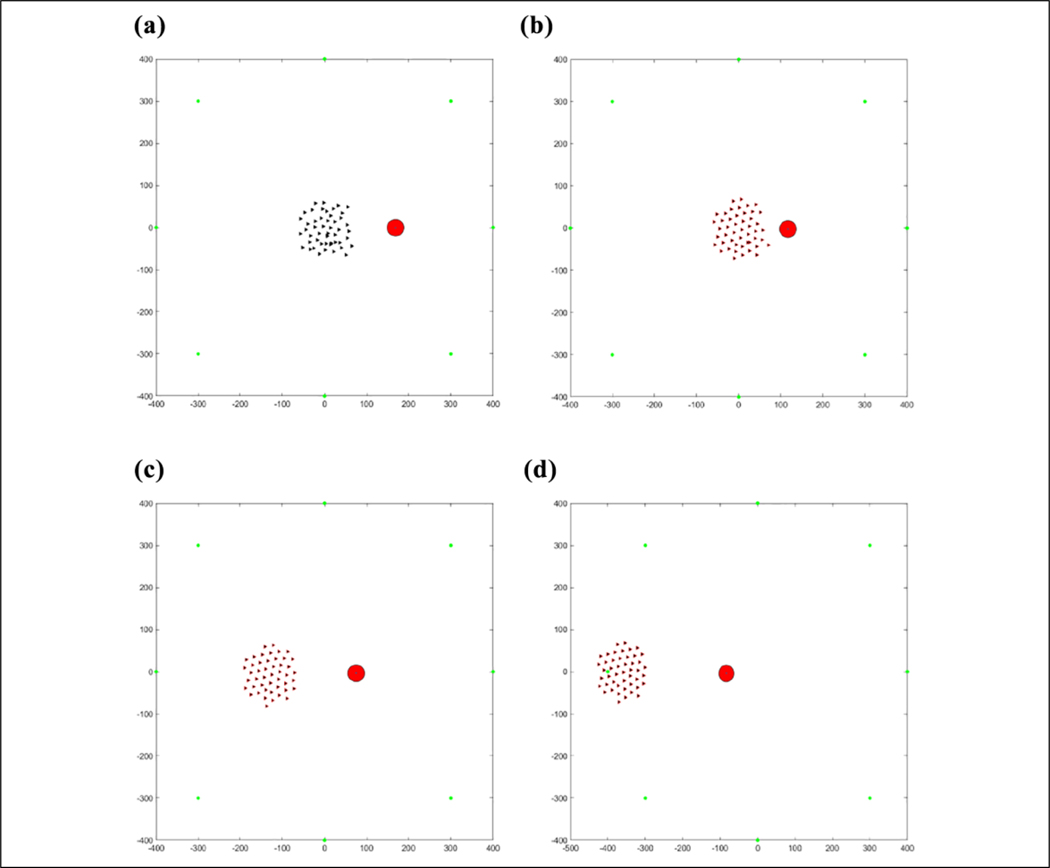
(a–d) Fifty agents flocking away from one predator after learning the same target.

The position of agents during the first learning episode can be seen in Figure 17. Some agents are in different states and the agents in the same state have not learned to go to the same target yet. This produces the messy flocking shape that can be seen. In Figure 18, the agents are all in the same state and have learned to go to the same target in an *α*-lattice formation.

**Figure 19.**
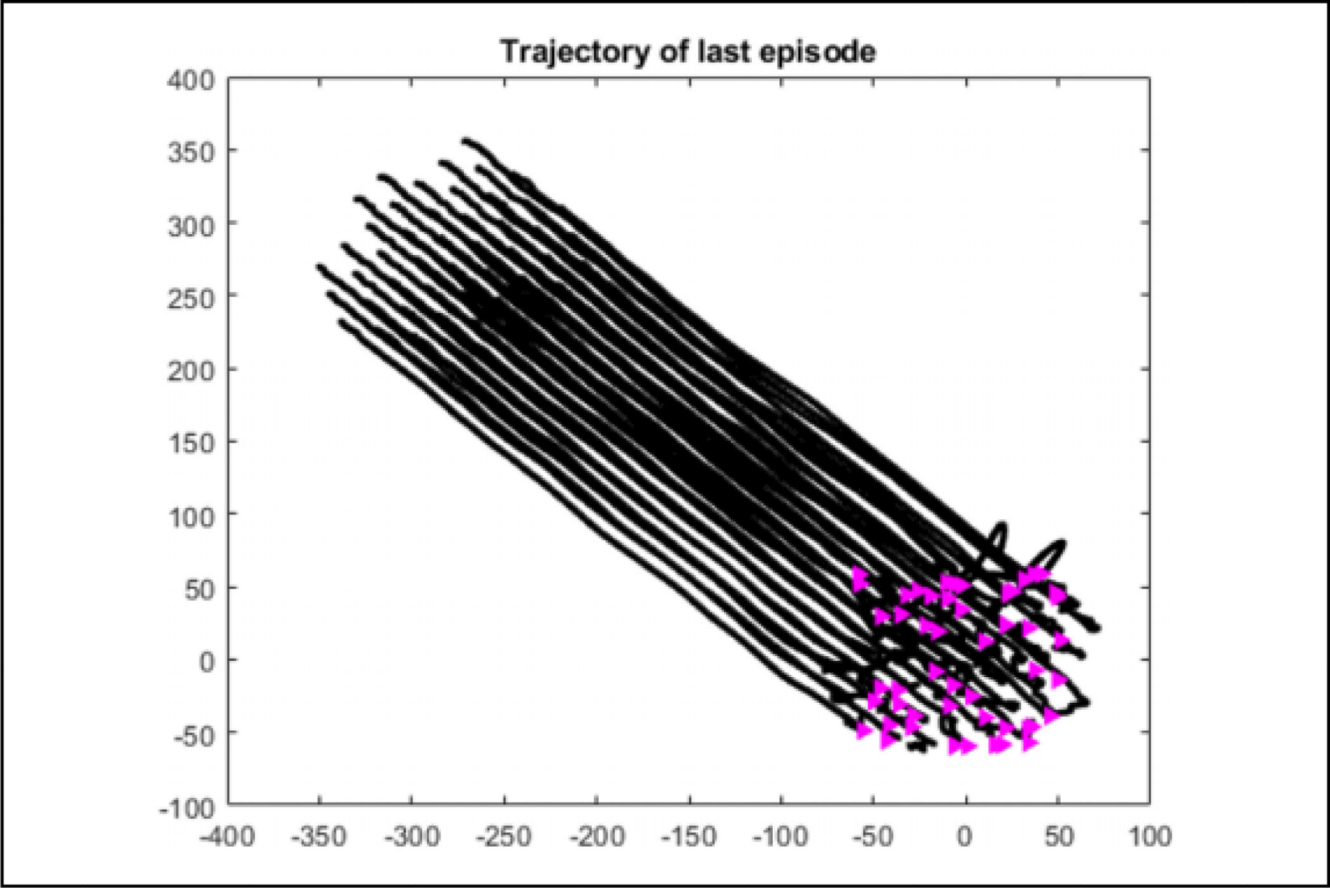
Trajectory the agents take in the last episode where the triangles are the initial position of the agents. The flocking can be seen to be smooth to the target after agents get into flocking formation despite the *ε*-greedy random action selection.

In Figure 19, we have the trajectory the agents take in the final learning episode, where the pink triangles are the random initialization of the agents. By the last episode, it can be seen that all agents have converged to the same target flocking in a relatively smooth manner despite the random action selection of *ε*-greedy.

#### Two predators.

**Figure 20.**
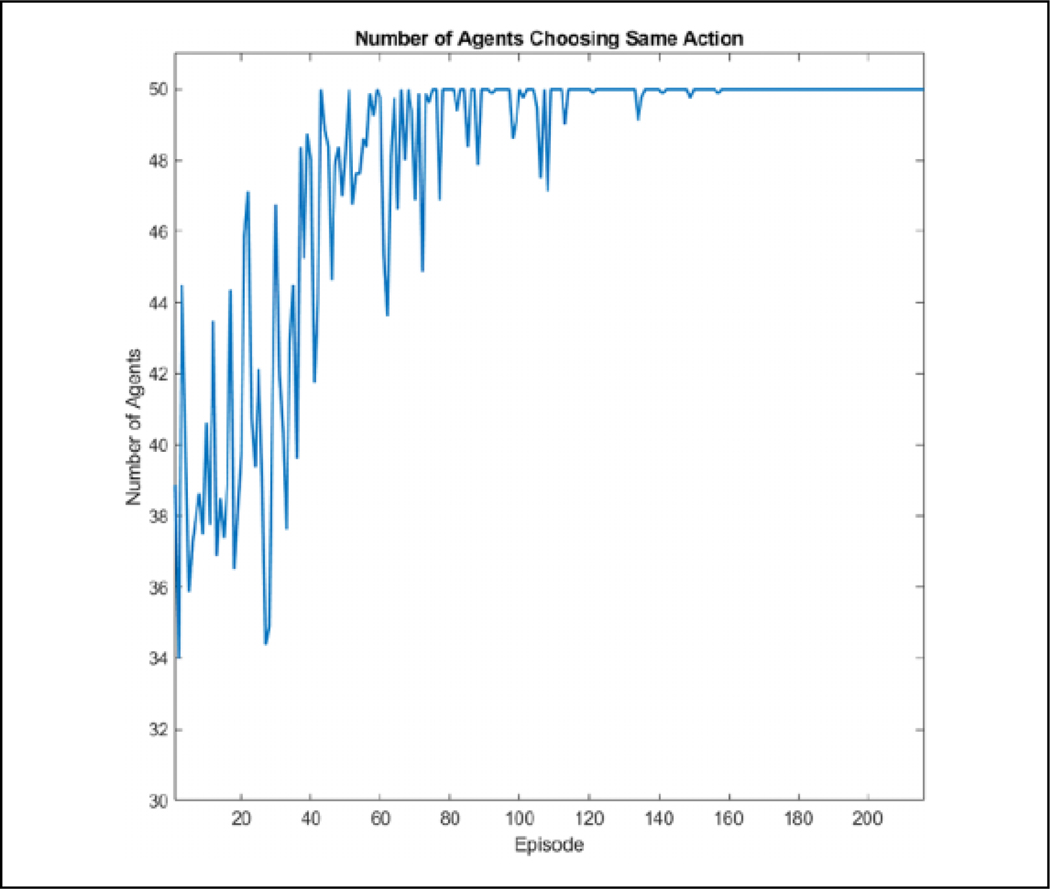
Number of agents choosing the same action for two predators over 216 episodes. It can be seen that after 180 episodes, all agents choose the same action for each state.

Two predators have also been tested to perform well with an expanded information vector to account for the extra predator and thus a larger state space as well. The addition of a second predator increases the number of predator starting positions by a factor of eight from 8 positions to 64. In addition to longer computation times for handling a second predator, there is now eight times the amount of episodes that must be performed for all direction combinations to be encountered. Unfortunately, this cannot be reduced, without reducing the problem size, by applying a function approximation approach to the learning process. However, there is a way to lower the amount of direction combinations. Instead of learning the direction of the two predators separately, we treat both predators as the same predator. This way if the first predator is detected in direction 1 and the second predator is detected in direction 2, it is the same as if the first predator is in direction 2 and the second predator is in direction 1. Thus, the system learns two state combinations simultaneously. However, there are eight direction combinations, where both predators are detected in the same direction, which is not reducible. This reduces the number of direction combinations from 64 down to 36, which reduces the number of episodes and thus the time it takes to learn from 8 times that of a single predator to 4.5 times. For this reason, the two-predator learning is done over 216 episodes, and the results of which can be seen in one run in Figure 20. It can be seen that by 144 episodes, or all combinations of directions being encountered four times the agents have almost converged. By 180 episodes or each direction being seen five times, the agents have completely converged to the same action for each state.

**Figure 21.**
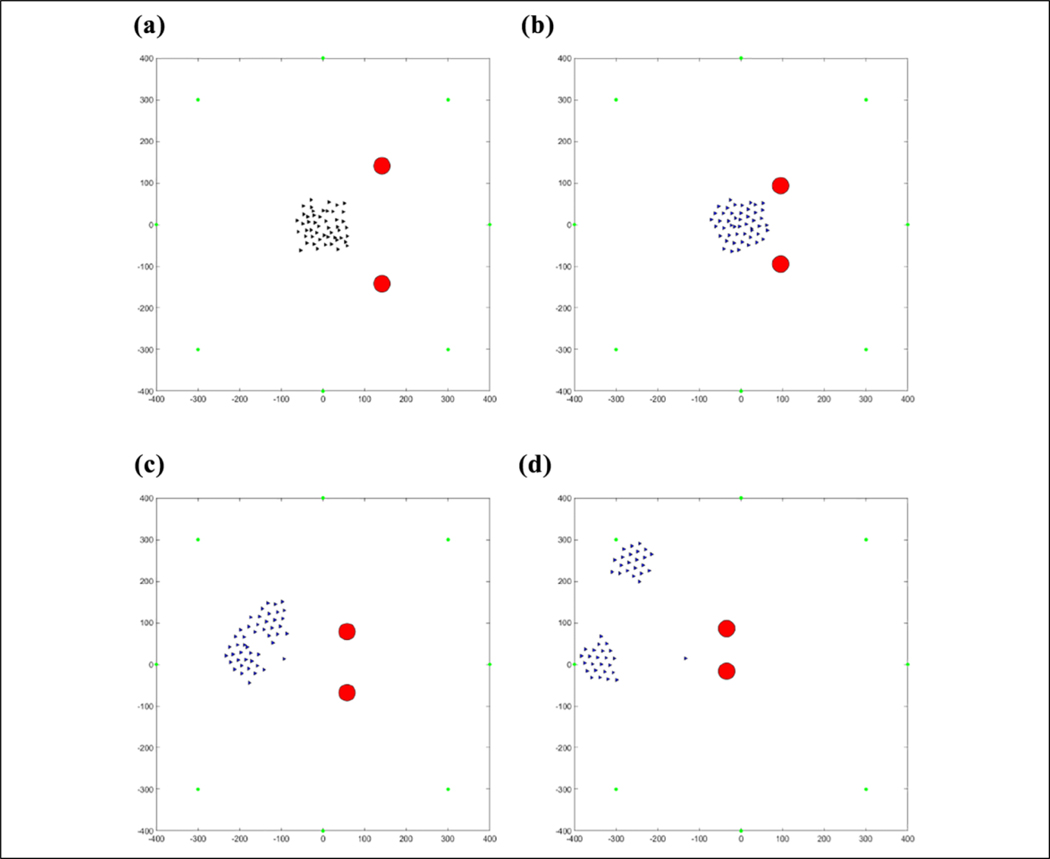
(a–d) Fifty agents flocking away from two predators before learning the same target.

**Figure 22.**
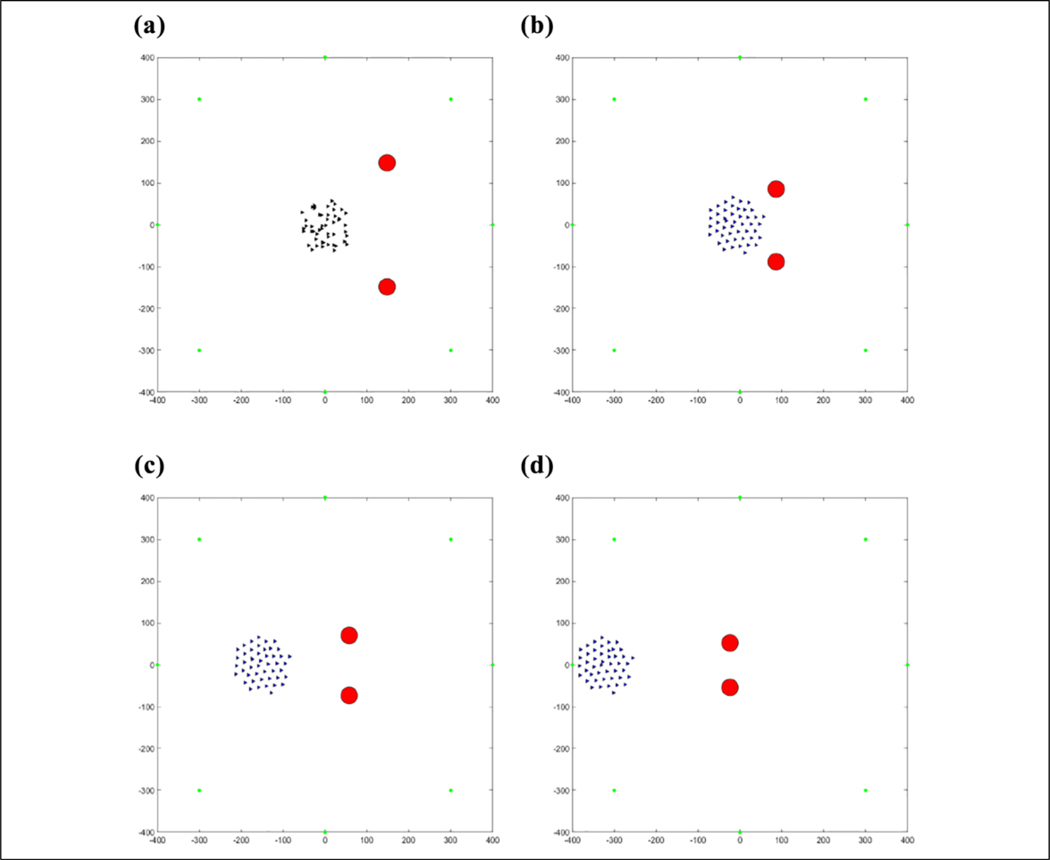
(a–d) Fifty agents flocking away from two predators after learning the same target.

The movement of the agents flocking away from the two predators before they have learned and after they have learned to flock away from the predators can be seen in Figures 21 and 22, respectively, which looks similar to that of the single predator.

### Discussion

In Figure 21, it can be seen that the agents split into two subgroups. This was a fairly common occurrence for unlearned episodes caused by the communication between neighbors. In this case, a lot of the agents may have found the west direction to be the best while others may have found the northwest direction to be best. Eventually, their close neighbors would agree with them and move with them to those targets but not enough to cause the other majority to agree. In this case, the neighborhoods are pretty split between the top half and the bottom half visually with agents on the border picking one or the other depending on the *ε*-greedy learning algorithm. The best direction is then learned in later episodes when the agents are randomly positioned so that the neighborhoods become mixed together.

Another aspect to consider is what would happen if agents were surrounding a predator. In particular, if agents were equally distributed around the predator, then the predator is not in any particular cardinal direction relative to the flock. Currently, there is no check for that occurring but that would be easily adaptable to stop the agents from moving until the predator moves into a position, where there is a cardinal direction. Alternatively, currently, the agents will move in either the first or last direction depending on implementation at which point the predator will likely obtain a direction after one time step.

## Conclusion and future work

### Conclusion

This article presented a hybrid system that achieves an efficient cooperative learning behavior. The system is applied to the task of escaping attacking predators while maintaining a flocking formation.

Flocking is used to move the agents in an *α*-lattice formation to a target location. Then, multiple reinforcement learning methods were presented utilizing the flocking targets as actions in the learning algorithm. Independent learning with and without function approximation proved to be unreliable in learning to flock together to the same target. Cooperative learning without function approximation was shown to be better than both independent learning methods but still took a considerable amount of episodes to learn. Cooperative learning with function approximation was shown to perform the best, requiring very few episodes for the agents to converge to the same target.

Finally, a method of detecting predators is used to determine the direction of attacking predators. The method proposed was shown to be very accurate, requiring only a few more iterations than it takes to transmit information from one edge of the flock to the other. The direction of the detected predators is used for determining the state of the system for the reinforcement learning component.

The hybrid system was then developed consisting of flocking control, function approximated cooperative learning, and consensus to allow agents to learn the location of a predator and where to flock away from it. The system was tested in one and two predator environments with results showing the success of the system as an efficient cooperative learning method.

### Future work

Although the hybrid-system proposed here was used to solve the task of avoiding predators by agents flocking together to targets, it is generic enough to be used in a variety of multiagent tasks. Applying this hybrid system of using consensus to determine the states and reinforcement learning to learn the states is something that can be looked further into in the future to achieve efficient learning for a variety of tasks. A possible improvement to this work is to create a more parallelized framework for the simulation to allow for faster learning particularly for increased amounts of predators that grow the state space and learning configuration. Further testing can be done with an expanded state size of more predators and a three-dimensional simulation environment. In addition to this, implementing smarter predators and different types of agents are also tasks that could be looked into in the future.

## Figures and Tables

**Table 1.** The iterations to converge and number of incorrect directions found over 900 iterations. *c*_*m*_ = 2 produces the best results.

Effects of change in *c*_*m*_						

	*c*_*m*_ = 0	*c*_*m*_ = 1	*c*_*m*_ = 2	*c*_*m*_ = 3	*c*_*m*_ = 4	*c*_*m*_ = 10

Iterations to converge	8.2	8.8	9.8	10.6	11	16.2
Incorrect directions	114 (12.7%)	75.2 (8.4%)	68.6 (7.6%)	73.8 (8.2%)	70.4 (7.8%)	54.4 (6.04%)
